# Protective Effects of Limonene and a Nano-Liposomal Limonene Formulation Against Chlorfenapyr-Induced Renal Toxicity: Mechanistic Insights into NRF2/HO-1 and NF-κB/COX-2 Signaling, Mitochondrial Dysfunction, and Apoptosis in Rat Kidneys

**DOI:** 10.3390/toxics14080668

**Published:** 2026-07-28

**Authors:** Ekramy M. Elmorsy, Amina A. Farag, Amal M. Abdel-Kareim, Marwa M. M. Fawzy, Mohammed A. Gebba, Samar Fawzy Gad, Nashwa E. Ahmed, Shimaa K. Mohamed, Hamada S. Salem, Ebtssam Beder, Sahar Soliman

**Affiliations:** 1 Center for Health Research, Northern Border University, Arar 73213, Saudi Arabia; ekramy.elmorsy@nbu.edu.sa; 2 Department of Forensic Medicine & Clinical Toxicology, Faculty of Medicine, Benha University, Benha 13518, Egypt; amina.farag@fmed.bu.edu.eg (A.A.F.); marwa.fawzy@fmed.bu.edu.eg (M.M.M.F.); 3 Department of Zoology, Faculty of Science, Benha University, Benha 13518, Egypt; amel.abdelkarim@fsc.bu.edu.eg; 4 Department of Anatomy & Embryology, Faculty of Medicine, Benha University, Benha 13518, Egypt; mohamed.gaba@fmed.bu.edu.eg (M.A.G.); samar.gad@fmed.bu.edu.eg (S.F.G.); 5 Department of Oral Surgery and Diagnostic Sciences, Faculty of Dentistry, Applied Science Private University, Amman 11937, Jordan; 6 Department of Medical Biochemistry and Molecular Biology, Faculty of Medicine, Benha University, Benha 13518, Egypt; nashwa.mohamed@fmed.bu.edu.eg; 7 Department of Pharmacology and Toxicology, Faculty of Pharmacy, Capital University, Cairo 11795, Egypt; shimaa_kamal@pharm.capu.edu.eg; 8 Department of Zoology, Faculty of Science, Mansoura University, Mansoura 35516, Egypt; hamada2012@mans.edu.eg; 9 Department of Forensic Medicine and Toxicology, Faculty of Veterinary Medicine, Benha University, Toukh 13736, Egypt; ebtssam.beder@fvtm.bu.edu.eg; 10 Department of Physiology and Pharmacology, College of Osteopathic Medicine, Sam Houston State University, Conroe, TX 77304, USA

**Keywords:** chlorfenapyr, limonene, liposomal nanoparticles, kidney injury, NRF2/HO-1, NF-κB/COX-2, apoptosis, oxidative stress, mitochondrial dysfunction

## Abstract

Chlorfenapyr (CFP) is a widely used pesticide associated with nephrotoxicity through oxidative stress, inflammation, and mitochondrial dysfunction. This study investigated the protective effects of limonene (LM) and its nano-liposomal formulation (LM-LNPs) against CFP-induced renal injury in rats. Animals were divided into six groups—control, LM, LM-LNPs, CFP, CFP + LM, and CFP + LM-LNPs—and treated orally for 30 days. CFP exposure resulted in marked renal dysfunction, histopathological and ultrastructural damage, suppression of the NRF2/HO-1/NQO1 antioxidant pathway, depletion of endogenous antioxidants excessive generation of reactive oxygen and nitrogen species, lipid peroxidation products, and DNA damage. These changes were accompanied by activation of NF-κB/COX-2-mediated inflammation, mitochondrial respiratory impairment, disrupted energy metabolism, and induction of apoptosis. Co-treatment with LM significantly ameliorated these alterations, whereas LM-LNPs produced greater improvements in renal function, tissue architecture, redox homeostasis, mitochondrial function, and inflammatory and apoptotic signaling. Immunohistochemical analyses further confirmed enhanced NRF2 expression and reduced NF-κB immunoreactivity in LM-LNP-treated kidneys. Overall, nano-liposomal delivery enhanced the renoprotective efficacy of limonene, highlighting its potential as a therapeutic strategy against pesticide-induced kidney injury.

## 1. Introduction

Chlorfenapyr (CFP; C_15_H_11_BrClF_3_N_2_O) is a pyrrole-class pro-insecticide widely used for the control of termites, malaria vectors, and a broad range of agricultural pests due to its unique mode of action and high insecticidal efficacy [1,2]. Despite its widespread application, growing evidence indicates that CFP poses substantial environmental and public health concerns. Residues have been detected in soil, water, and food commodities at measurable concentrations, reflecting its environmental persistence and potential for bioaccumulation [3,4,5]. Human intoxication with CFP is associated with a particularly high mortality rate, reaching up to 75% in reported cases [6]. Early manifestations of poisoning, including chest tightness, fatigue, and drowsiness, may rapidly progress to hyperthermia, excessive sweating, respiratory failure, and death [6,7,8].

Chlorfenapyr continues to be widely used because of its broad-spectrum insecticidal activity and effectiveness against insect populations resistant to conventional pesticides, despite the increasing global emphasis on integrated pest management and reducing pesticide use in agriculture. Consequently, cases of accidental, occupational, and intentional CFP poisoning continue to be reported worldwide and are associated with substantial morbidity and mortality despite advances in supportive medical care [9]. Although CFP poisoning is relatively uncommon, it carries a remarkably high fatality rate. A recent systematic review identified 75 published human cases, reporting an overall case fatality rate of approximately 76%, with survivors frequently experiencing persistent neurological sequelae [10].

The toxicity of CFP is largely attributed to its metabolic conversion to tralopyril, its principal active metabolite, in both target organisms and mammals [11,12]. Tralopyril acts as a potent uncoupler of oxidative phosphorylation by disrupting the mitochondrial proton gradient and impairing ATP production, ultimately leading to cellular energy depletion [4,11]. Increasing evidence indicates that CFP-induced mitochondrial dysfunction is accompanied by excessive reactive oxygen species (ROS) generation, activation of inflammatory pathways, and apoptotic cell death. In addition to its well-established hepatotoxic and enterotoxic effects [9], recent studies have demonstrated the accumulation of CFP and tralopyril in renal tissue, identifying the kidney as a major target organ of toxicity [13]. Nevertheless, the molecular mechanisms underlying CFP-induced nephrotoxicity, particularly those involving oxidative stress, inflammatory signaling, mitochondrial dysfunction, and apoptosis, remain incompletely understood. Elucidating these mechanisms and identifying effective therapeutic interventions are therefore of considerable toxicological and clinical importance.

Limonene (LM), a naturally occurring monocyclic monoterpene (1-methyl-4-(1-methylethenyl)cyclohexene), is a major constituent of citrus essential oils and accounts for more than 95% of the terpenoid content of orange peel oil [14]. Accumulating evidence has highlighted a broad spectrum of pharmacological activities associated with LM, including antioxidant, anti-inflammatory, anti-apoptotic, antidiabetic, and anticancer effects [15,16,17,18]. These protective properties have been attributed to its ability to enhance endogenous antioxidant defenses, activate cytoprotective pathways such as NRF2/HO-1, suppress NF-κB-mediated inflammatory signaling, and preserve mitochondrial integrity and function [19,20]. In addition, LM has demonstrated immunomodulatory, hypolipidemic, and hepatoprotective activities in various experimental models [21].

Despite its promising biological profile, the therapeutic application of LM is limited by several unfavorable physicochemical characteristics, including poor aqueous solubility, low oral bioavailability, high volatility, and rapid metabolic clearance [22,23]. Nanotechnology-based delivery systems have emerged as an effective strategy to overcome these limitations. Among these approaches, liposomal nanoparticles (LNPs) are particularly attractive owing to their excellent biocompatibility, structural resemblance to biological membranes, and high capacity for encapsulating lipophilic compounds [24,25]. Liposomal formulations can improve drug solubility and stability, prolong systemic circulation, enhance cellular uptake, and optimize tissue distribution [26,27,28]. Moreover, liposomal encapsulation has been shown to amplify the antioxidant and anti-inflammatory activities of several natural compounds, suggesting that a similar enhancement may be achieved with LM [29].

To date, the comparative renoprotective efficacy of free LM and its nanoliposomal formulation against CFP-induced renal injury has not been systematically investigated. Therefore, the present study aimed to evaluate the protective effects of LM and LM-loaded liposomal nanoparticles (LM-LNPs) in a rat model of CFP-induced nephrotoxicity, with particular emphasis on NRF2/HO-1-mediated antioxidant defense; NF-κB/COX-2-driven inflammatory signaling; mitochondrial dysfunction, including alterations in ATP production, pyruvate dehydrogenase activity, and electron transport chain complexes I-IV; and apoptosis-related pathways. We hypothesized that liposomal encapsulation would enhance the biological efficacy of LM and provide superior protection against CFP-induced renal damage. Our findings demonstrate that CFP induces severe renal injury characterized by oxidative stress, inflammation, mitochondrial dysfunction, and apoptotic cell death, whereas both LM and LM-LNPs significantly attenuate these pathological alterations. Notably, LM-LNPs afforded greater protection than free LM, resulting in more pronounced restoration of antioxidant defenses and mitochondrial function together with stronger suppression of inflammatory and apoptotic responses. Collectively, these findings identify mitochondrial dysfunction as a central mechanism underlying CFP-induced nephrotoxicity and highlight nanoliposomal delivery as a promising strategy for enhancing the therapeutic potential of limonene against pesticide-induced renal injury.

## 2. Materials and Methods

### 2.1. Preparation and Characterization of Limonene-Loaded Liposomal Nanoparticles (LM-LNPs)

Limonene-loaded liposomal nanoparticles (LM-LNPs) were prepared using the thin-film hydration method followed by probe sonication, as previously described with minor modifications [27]. The formulation was characterized by determining particle size, polydispersity index (PDI), zeta potential, morphology, encapsulation efficiency (EE), drug loading (DL), the in vitro release profile, and Fourier transform infrared (FTIR) spectra to confirm successful encapsulation and evaluate its physicochemical properties. Limonene content for the determination of encapsulation efficiency and in vitro release was quantified by UV spectrophotometry at 210 nm using a validated calibration curve. Appropriate blank liposomal formulations were included to minimize potential background interference during quantification. Full details of the formulations, analytical methodologies, calculation equations, characterization procedures, and experimental data are provided in the Supplementary Materials (Methods S1–S4).

### 2.2. Experimental Animals

Sixty adult male Wistar rats were obtained from the Medical Experimental Research Center (MERC), Faculty of Medicine, Mansoura University, Egypt. At study initiation, the animals had a mean body weight of 164.28 ± 9.50 g. They were housed in standard polypropylene cages under controlled laboratory conditions maintained at 25 ± 2 °C and 45 ± 5% relative humidity, with a 12 h light/dark cycle. Standard rodent chow and water were provided ad libitum throughout the study. The animals underwent a two-week acclimatization period prior to treatment initiation to minimize stress-related variability and permit assessment of their baseline health status.

### 2.3. Ethical Compliance

All experimental procedures were conducted in accordance with applicable institutional and international guidelines governing the care and use of laboratory animals. The experimental protocol was approved by the Institutional Animal Care and Use Committee (IACUC) of Mansoura University [Approval No. MU-ACUC (SC.R.26.05.44)].

### 2.4. Inclusion and Exclusion Criteria

Inclusion criteria. Animals eligible for inclusion were healthy adult male Wistar rats that displayed normal general health and typical locomotor activity throughout the acclimation period, with no clinical signs of illness, injury, or disease at the time of treatment initiation.

Exclusion criteria. Animals displaying aberrant clinical symptoms, congenital anomalies, or evidence of disease at any point before treatment initiation were excluded from the study prior to randomization. No animals meeting these exclusion criteria were identified; consequently, all animals that underwent randomization were retained throughout the study duration, and none were excluded following treatment allocation.

### 2.5. Experimental Design and Treatment Protocol

Animals were randomly allocated into six experimental groups (*n* = 10 per group). Group I (Control) received corn oil by oral gavage as the vehicle control; corn oil was selected because it is pharmacologically inert at the administered volume and has no reported nephrotoxic effects. Group II (LM) received free limonene (Sigma-Aldrich, St. Louis, MO, USA) at a dose of 100 mg/kg body weight/day by oral gavage. Group III (LM-LNPs) received limonene-loaded liposomal nanoparticles, freshly dispersed in distilled water immediately before dosing and delivered by the same route at an equivalent limonene dose of 100 mg/kg body weight/day; this dose was selected on the basis of previous studies demonstrating its safety and suitability for repeated dosing [20]. Group IV (CFP) received chlorfenapyr (commercial formulation: Challenger Super^®^ 24% SC) at 180 mg/kg body weight/day, suspended in corn oil. This dose, corresponding to approximately 40% of the reported oral LD_50_ (441 mg/kg; JMPR, 2005), was selected to induce reproducible toxicological alterations while minimizing mortality, thereby allowing assessment of potential protective interventions [30]. Groups V (CFP + LM) and VI (CFP + LM-LNPs) received chlorfenapyr in combination with free limonene or LM-LNPs, respectively, at the doses described above.

All treatments were given once daily by oral gavage for 30 consecutive days. The oral route was chosen because it closely reflects the principal routes of environmental and dietary pesticide exposure in humans, thereby enhancing the physiological relevance of the experimental model. In the combination groups, chlorfenapyr was given at least 30 min before limonene or LM-LNPs to minimize the potential for direct physicochemical interaction within the gastrointestinal tract, ensuring that any protective effects observed would reflect the biological activity of limonene rather than chemical neutralization of chlorfenapyr. No humane endpoints requiring early euthanasia were reached, and no mortality occurred in any group during the 30-day treatment period (mortality rate = 0%).

### 2.6. Tissue Collection and Processing

Animals were fasted for 10 h prior to sacrifice to minimize metabolic variability. At the end of the experimental period, rats were anesthetized with isoflurane, and adequate anesthesia was confirmed by the absence of reflex responses. Blood samples were then collected by cardiac puncture into plain tubes. Euthanasia was subsequently performed using an overdose of isoflurane (≥5% in oxygen) administered for at least 5 min following loss of consciousness, in accordance with established ethical guidelines for laboratory animal research. Death was confirmed by the absence of cardiac and respiratory activity as well as corneal and pedal reflexes. Blood samples were allowed to clot at room temperature for 20–30 min. Serum was separated by centrifugation at 3000 rpm for 10 min at 4 °C, aliquoted, and stored at −80 °C until analysis.

To minimize allocation bias, animals were randomly assigned to subsequent analyses: seven animals per group were allocated for biochemical and molecular investigations, and three animals per group were reserved for histopathological and ultrastructural examinations. All animals were followed through to study completion and included in the final analyses, with no data points excluded from any outcome assessment.

Following blood collection, both kidneys were excised, rinsed with ice-cold normal saline to remove residual blood, and divided into separate portions according to the intended analysis. Samples designated for biochemical assays were snap-frozen in liquid nitrogen and stored at −80 °C until use. Kidney tissues intended for gene-expression analysis were either preserved in RNA stabilization solution or immediately frozen to prevent RNA degradation. Tissues designated for histopathological and immunohistochemical examinations were fixed in 10% neutral-buffered formalin. Samples intended for ultrastructural examination were fixed separately in 2.5% glutaraldehyde prepared in 0.1 M phosphate buffer and processed for transmission electron microscopy as detailed in Supplementary Methods S5.

For biochemical and protein-based analyses, renal tissue homogenates were prepared in phosphate-buffered saline (10 mM, pH 7.4) containing protease inhibitors and centrifuged at 4000 rpm for 10 min at 4 °C. The resulting supernatants were collected and stored at −70 °C until analysis.

### 2.7. Assessment of Serum Biochemical Markers of Renal Function

Serum creatinine, urea, and uric acid concentrations were measured using approved commercial colorimetric assay kits (Diagnostic, Giza, Egypt) in accordance with the manufacturer’s instructions to evaluate renal function. Relevant assay specifications are provided in Supplementary Table S1.

### 2.8. Assessment of Renal Oxidative Stress and Antioxidant Status

Renal oxidative stress and antioxidant status were evaluated by measuring reduced glutathione (GSH), malondialdehyde (MDA), and the activities of superoxide dismutase (SOD) and catalase (CAT) using commercial colorimetric assay kits (BioDiagnostic, Giza, Egypt); protein carbonyl (PC) and reactive oxygen species (ROS) using commercial assays (MyBioSource, San Diego, CA, USA); and nitric oxide (NO) using an established colorimetric method. Renal HO-1 and NRF2 protein levels were quantified using ELISA kits (Abcam, Cambridge, UK). All commercial assays were performed according to the manufacturers’ instructions. Relevant assay specifications are provided in Supplementary Table S1.

### 2.9. Assessment of Apoptosis-Related and Mitochondrial Proteins

Renal apoptosis was assessed by measuring caspase-3, Bax, and Bcl-2 protein levels using commercial ELISA kits (MyBioSource, San Diego, CA, USA), whereas mitochondrial integrity was evaluated by determining cytochrome c concentrations using a commercial ELISA kit (Elabscience Biotechnology Inc., Houston, TX, USA), according to the manufacturers’ instructions. Relevant assay specifications are provided in Supplementary Table S1.

### 2.10. Assessment of Pro-Inflammatory Cytokines and NF-κB Activation

Renal inflammatory status was evaluated by quantifying the levels of TNF-α, IL-1β, and IL-6 using commercial ELISA kits (Assay Genie, Dublin, Ireland) and NF-κB p65 using a SimpleStep ELISA kit (Abcam, Cambridge, UK), in accordance with the manufacturers’ instructions. Relevant assay specifications are provided in Supplementary Table S1.

### 2.11. Assessment of DNA Fragmentation

Apoptotic DNA fragmentation in kidney tissue was determined using a commercial ELISA kit (MyBioSource, San Diego, CA, USA) according to the manufacturer’s protocol. Relevant assay specifications are provided in Supplementary Table S1.

### 2.12. RNA Isolation and RT-qPCR Analysis

Total RNA was extracted from kidney tissue and complementary DNA (cDNA) was produced with conventional commercial reagents. Using a real-time PCR apparatus (Table S2) and gene-specific primers, quantitative real-time PCR (RT-qPCR) with SYBR Green chemistry was carried out. The comparative 2^−ΔΔCt^ approach developed by Livak and Schmittgen was used to calculate relative gene expression after normalization to the housekeeping gene [31]. Full details of the RNA extraction procedure, reagents, thermal cycling conditions, and primer sequences are provided in the Supplementary Materials (Methods S6).

### 2.13. Assessment of Mitochondrial Respiratory Chain Complex Activities

The activities of mitochondrial electron transport chain complexes I-IV were determined spectrophotometrically by established methods as stated earlier [32,33]. Enzyme activities are reported as μmol/min/mg protein and adjusted to the total protein content. Methods S7 provides specifics on the reaction mixtures and experimental conditions employed.

### 2.14. Assessment of Cellular Energy Metabolism

Renal cellular energy metabolism was evaluated by measuring ATP concentration using an ATP Assay Kit (Jiancheng Bioengineering Institute, Nanjing, China) and pyruvate dehydrogenase (PDH) activity using an enzyme activity assay (Solarbio, Beijing, China), according to the manufacturers’ instructions. Relevant assay specifications are provided in Supplementary Table S1.

### 2.15. Histopathological and Ultrastructural Examination

Kidney samples were processed for routine histological examination. Paraffin-embedded tissues were sectioned at a thickness of 5 µm and stained with hematoxylin and eosin (H&E). Renal histopathological alterations were evaluated using a predefined semiquantitative scoring system assessing tubular injury and necrotic changes, glomerular alterations, inflammation, and hemorrhage, as detailed in Table S3. Following standard fixation, embedding, sectioning, and staining, ultrastructural alterations were examined using transmission electron microscopy (JEM-2100; JEOL Ltd., Tokyo, Japan). Detailed descriptions of the TEM procedures, scoring criteria, and tissue processing protocols are provided in the Supplementary Materials (Methods S5). Histopathological scoring and ultrastructural evaluation were performed using coded samples by investigators blinded to treatment allocation.

### 2.16. Immunohistochemical Assay of NRF2 and NF-κB

Using a horseradish peroxidase (HRP)-based detection technique, paraffin-embedded kidney sections were stained immunohistochemically for NRF2 and NF-κB p65 using validated primary antibodies. Immunoreactivity was observed using 3,3′-diaminobenzidine (DAB) and ImageJ software (version 1.54s; National Institutes of Health, Bethesda, MD, USA) was used to quantify positively stained regions. Detailed immunohistochemical staining procedures, antibody information, incubation conditions, and image analysis protocols are provided in the Supplementary Materials (Methods S8). Immunohistochemical image analysis was performed using coded samples by investigators blinded to treatment allocation.

### 2.17. Statistical Analysis

Data were analyzed utilizing SAS software (Version 9.4; SAS Institute Inc., Cary, NC, USA). Normality and homogeneity of variance were evaluated through the Shapiro–Wilk and Levene’s tests, respectively. Differences among experimental groups were assessed using one-way analysis of variance (ANOVA), with Tukey’s honestly significant difference (HSD) post hoc test applied for multiple comparisons when significant effects were identified.

Results are expressed as mean ± standard error of the mean (SEM). A *p*-value of less than 0.05 was considered statistically significant. Graphs were generated using GraphPad Prism version 10.4.1 (GraphPad Software, Boston, MA, USA).

## 3. Results

### 3.1. Physicochemical Characterization of LM-LNPs

**Figure 1:**
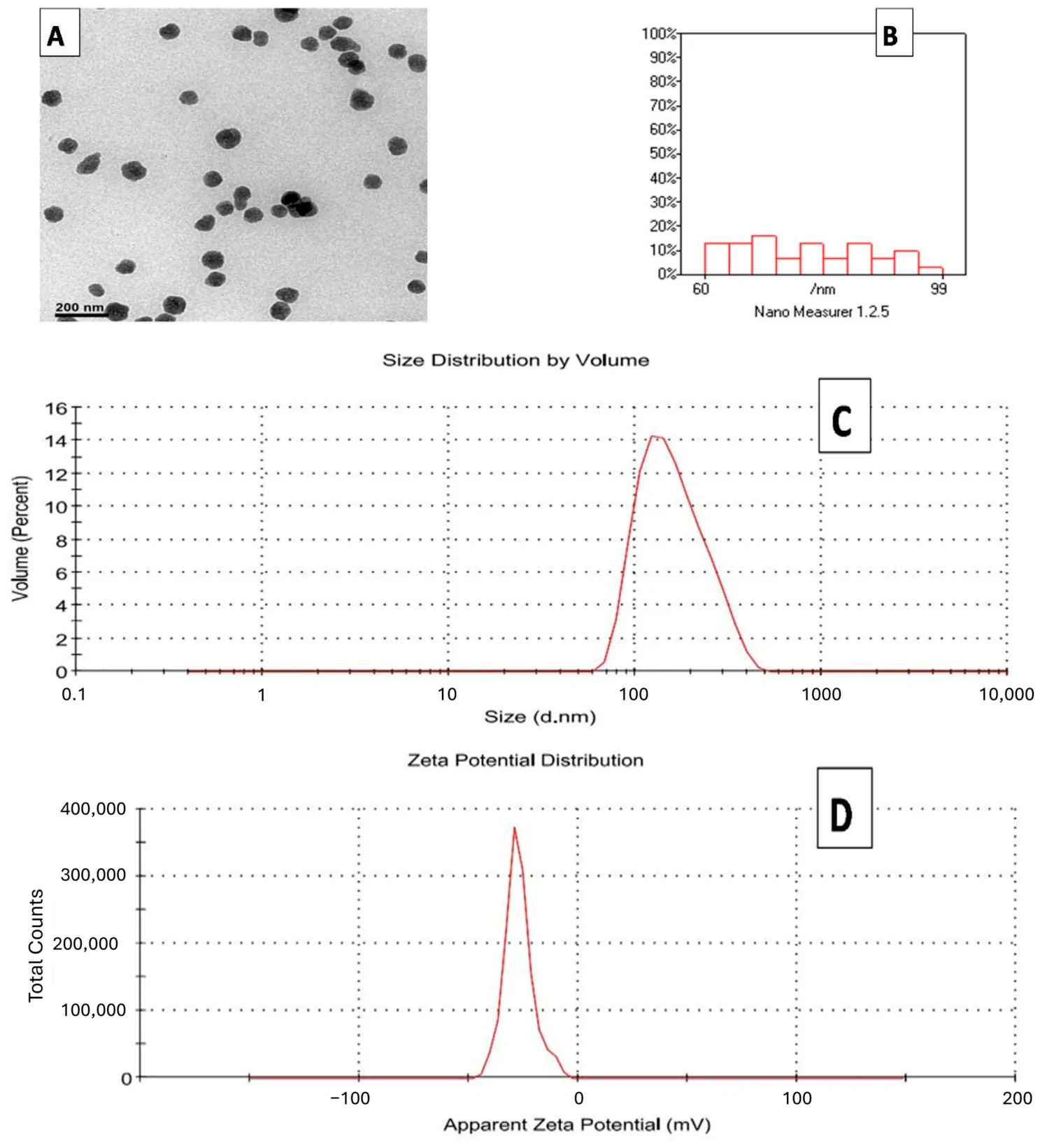
(**A**) TEM image of LM-LNPs showing spherical morphology. (**B**) Size distribution mainly between 60–99 nm. (**C**) Volume-based size distribution. (**D**) Zeta potential distribution.

Transmission electron microscopy (TEM) revealed that LM-LNPs exhibited a spherical morphology with a uniform size distribution and minimal aggregation, indicating successful nanoparticle formation (Figure 1A). Consistent with these observations, dynamic light scattering (DLS) analysis demonstrated a narrow particle size distribution ranging from 60 to 99 nm (Figure 1B). The mean hydrodynamic diameter of LM-LNPs was 165 nm, reflecting the hydrated particle size measured in suspension. The formulation displayed a low polydispersity index (PDI) of 0.165, indicating excellent size homogeneity and colloidal stability (Figure 1C). Furthermore, LM-LNPs exhibited a zeta potential of −38.10 mV, suggesting a highly stable nanosystem with strong electrostatic repulsion between particles, thereby minimizing aggregation and promoting dispersion stability (Figure 1D).

### 3.2. Encapsulation Efficiency and Drug Loading of LM-LNPs

The encapsulation efficiency and drug loading of LM-LNPs were determined by quantifying free limonene in the supernatant using UV-Vis spectrophotometry at 210 nm. The formulation exhibited a high encapsulation efficiency of 85.31 ± 2.15%, indicating effective incorporation of limonene within the lipid bilayer. This high entrapment efficiency is likely attributable to the hydrophobic nature of limonene and its strong affinity for the lipid matrix. The drug loading was 8.10 ± 0.42%, reflecting efficient incorporation of limonene relative to the total lipid content. Collectively, these findings confirm the successful preparation of a stable liposomal formulation with high encapsulation capacity.

### 3.3. FTIR Characterization of Crude LM, Blank Liposomes, and LM-LNPs

**Figure 2:**
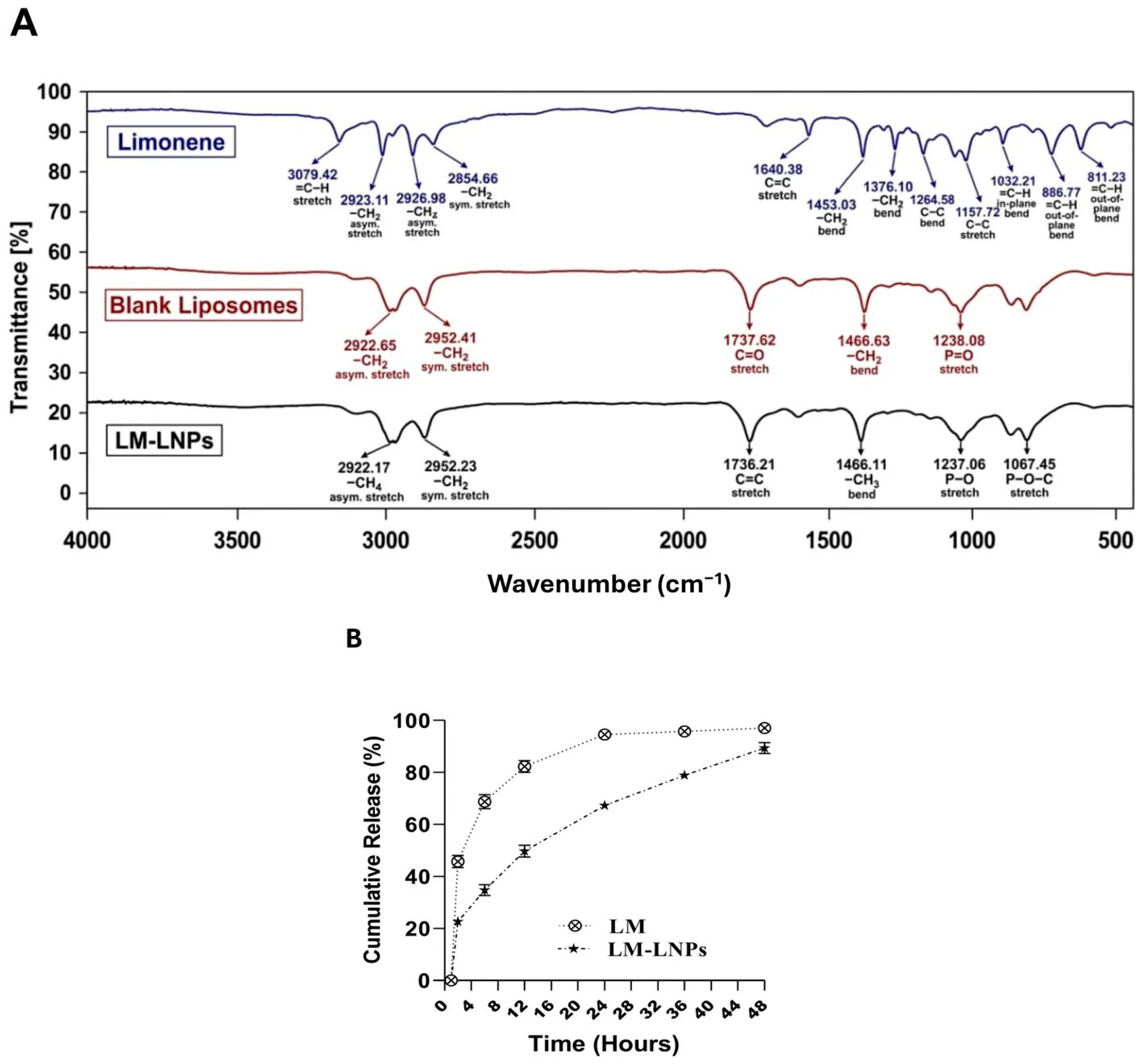
(**A**) FTIR spectra of limonene, blank liposomes, and LM-LNPs. The retention of characteristic limonene and lipid absorption bands with minor spectral shifts confirms successful encapsulation of limonene within the liposomal matrix without chemical modification. (**B**) In vitro release profiles of free limonene and LM-LNPs. Free limonene exhibited rapid release, whereas LM-LNPs showed a sustained and controlled release pattern, demonstrating the ability of liposomal encapsulation to prolong limonene release and improve drug retention.

FTIR analysis was performed to confirm the successful incorporation of limonene into the liposomal formulation (Figure 2A). The spectrum of free limonene displayed characteristic absorption bands at 3079 cm^−1^ corresponding to =C-H stretching vibrations, as well as prominent peaks at 2963 and 2854 cm^−1^ attributed to aliphatic C-H stretching. A distinct band at 1640 cm^−1^ was observed due to C=C stretching of the alkene group. Blank liposomes exhibited characteristic phospholipid absorption bands, including peaks at 2922 and 2852 cm^−1^ corresponding to CH_2_ stretching vibrations and a prominent carbonyl (C=O) band at 1737 cm^−1^. In the LM-LNP spectrum, the major characteristic peaks of both limonene and the liposomal matrix were retained, with slight shifts in peak position and intensity. The preservation of these characteristic functional groups, together with minor spectral changes, indicates successful encapsulation of limonene within the lipid bilayer without evidence of chemical incompatibility or structural modification (Figure 2A).

### 3.4. Effect of Liposomal Encapsulation on the In Vitro Release Profile of Limonene

The in vitro release profiles of free limonene and LM-LNPs are presented in Figure 2B. Free limonene exhibited a rapid release pattern, with 44.9 ± 2.3% of the drug released within the first 2 h. The cumulative release increased to 69.5 ± 2.57% at 6 h and exceeded 90% after 24 h, reaching 97.1 ± 1.12% at 48 h, indicative of an immediate and uncontrolled diffusion profile. In contrast, LM-LNPs displayed a sustained release behavior characterized by an initial burst release of 21.8 ± 1.78% at 2 h, followed by a gradual and controlled release over time. The cumulative release reached 33.98 ± 2.10% at 6 h, 48.57 ± 2.50% at 12 h, 67.34 ± 2.01% at 24 h, and 88.34 ± 2.53% at 48 h. These findings demonstrate that liposomal encapsulation effectively prolonged limonene release and enhanced drug retention compared with the free compound, confirming the sustained-release properties of the LM-LNP formulation (Figure 2B).

### 3.5. Effect of Limonene and LM-LNPs on Chlorfenapyr-Induced Renal Dysfunction

**Figure 3:**
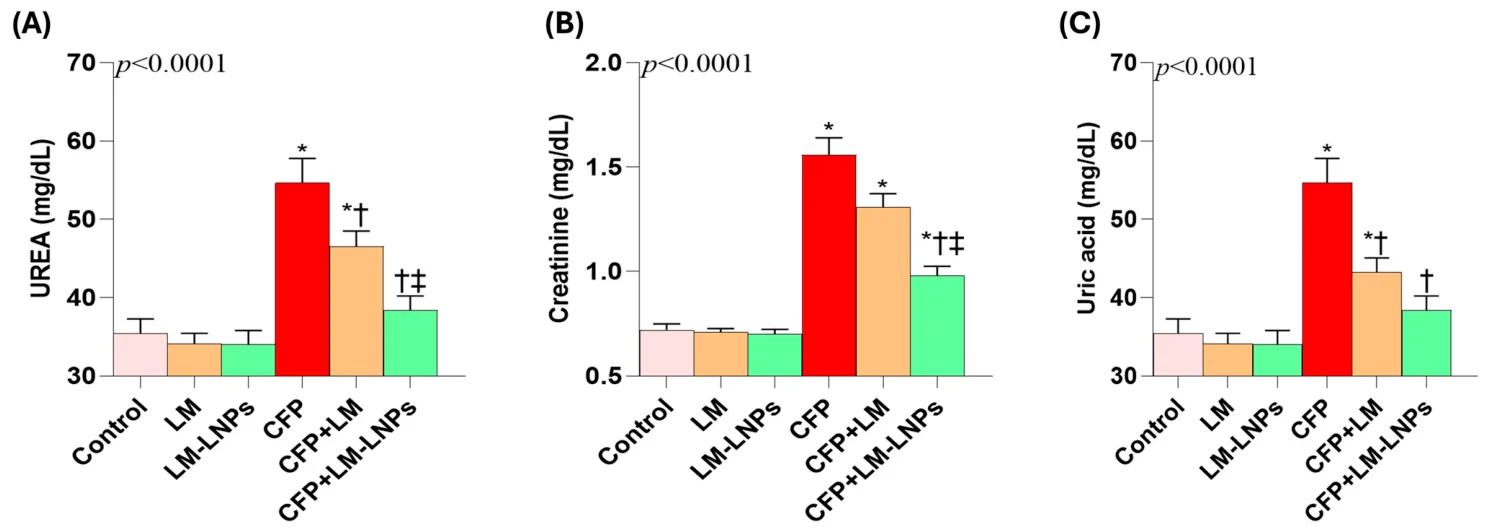
Protective effects of limonene (LM) and limonene-loaded liposomal nanoparticles (LM-LNPs) against chlorfenapyr (CFP)-induced alterations in serum renal function markers. (**A**) Urea, (**B**) creatinine, and (**C**) uric acid levels. Data are presented as mean ± SEM (*n* = 7). Symbols denote significant differences (*p* < 0.05): * versus Control, † versus CFP, and ‡ versus CFP + LM.

Chlorfenapyr (CFP) administration induced marked renal dysfunction, as evidenced by significant elevations in serum urea, creatinine, and uric acid levels compared with the control group (Figure 3A–C). Serum urea increased by approximately 57%, while creatinine and uric acid levels increased by approximately 117% and 107%, respectively, confirming substantial impairment of renal function.

Co-administration of limonene (LM) partially ameliorated these alterations. Compared with the CFP-treated group, LM reduced serum urea, creatinine, and uric acid levels by approximately 17%, 17%, and 20%, respectively. In contrast, LM-loaded liposomal nanoparticles (LM-LNPs) produced markedly greater renoprotective effects, reducing serum urea by approximately 29%, creatinine by 37%, and uric acid by 39% relative to the CFP group.

Notably, the LM-LNP formulation restored serum uric acid levels to values approaching those of the control group and produced a substantially greater improvement in all measured renal function markers than free LM. These findings indicate that liposomal encapsulation significantly enhanced the renoprotective efficacy of limonene against CFP-induced kidney injury.

### 3.6. Effect of Limonene and LM-LNPs on Chlorfenapyr-Induced Alterations in NRF2 Signaling, Antioxidant Defenses, Oxidative Stress, and DNA Damage

**Figure 4:**
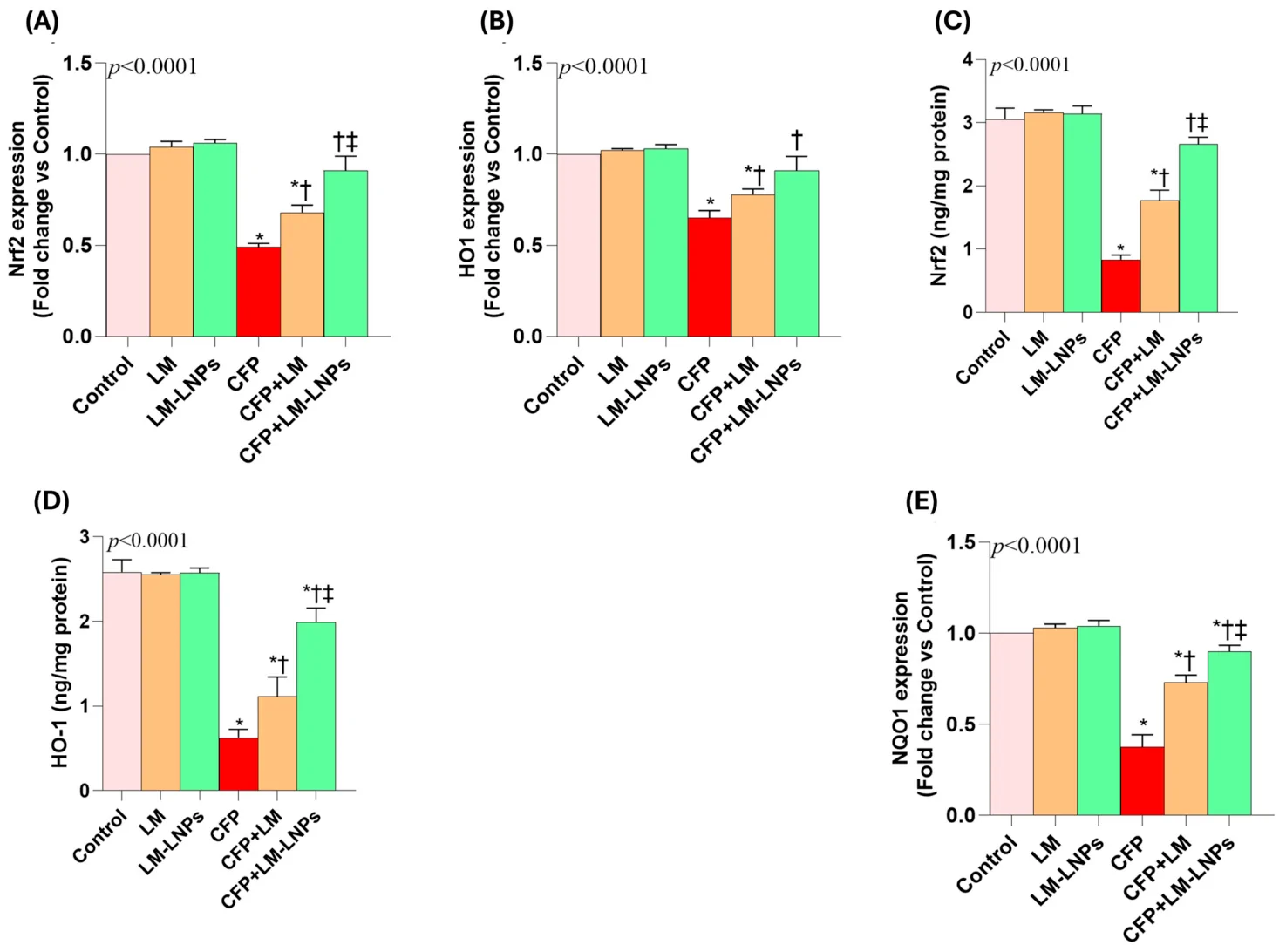
Protective effects of limonene (LM) and limonene-loaded liposomal nanoparticles (LM-LNPs) against chlorfenapyr (CFP)-induced alterations in the NRF2 antioxidant signaling pathway. (**A**) *NRF2* (nuclear factor erythroid 2-related factor 2) gene expression, (**B**) *HO-1* (heme oxygenase-1) gene expression, (**C**) NRF2 protein level, (**D**) HO-1 protein level, and (**E**) *NQO1* (NAD(P)H quinone dehydrogenase 1) gene expression. Data are presented as mean ± SEM (*n* = 7). Symbols denote significant differences (*p* < 0.05): * versus Control, † versus CFP, and ‡ versus CFP + LM.

Chlorfenapyr (CFP) exposure markedly impaired the NRF2/HO-1/NQO1 antioxidant signaling pathway, as evidenced by significant reductions in both gene and protein expression levels compared with the control group (Figure 4A–E). *NRF2*, *HO-1*, and *NQO1* gene expression decreased by approximately 50%, 35%, and 62%, respectively. Likewise, NRF2 and HO-1 protein levels were reduced by approximately 73% and 78%, respectively, indicating profound suppression of endogenous antioxidant defense mechanisms. Co-treatment with limonene (LM) partially restored NRF2 pathway activity, whereas LM-loaded liposomal nanoparticles (LM-LNPs) produced a substantially greater effect. Compared with the CFP group, LM-LNPs increased *NRF2*, *HO-1*, and *NQO1* expression by approximately 82%, 38%, and 145%, respectively, while NRF2 and HO-1 protein levels increased by approximately 188% and 209%, respectively. Notably, *NRF2* and *HO-1* gene expression in the LM-LNP-treated group approached control values, demonstrating effective restoration of antioxidant signaling.

**Figure 5:**
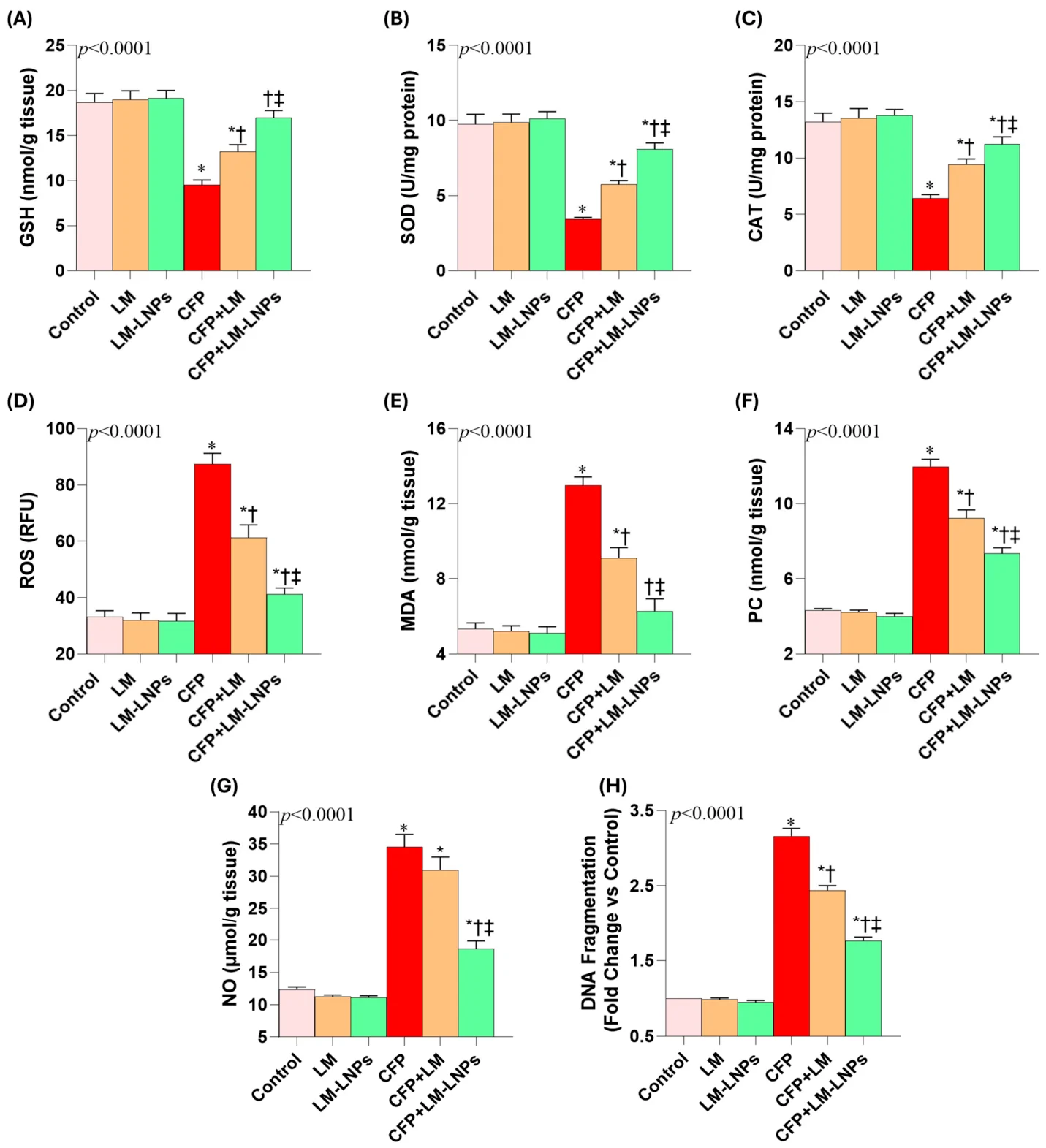
Protective effects of limonene (LM) and limonene-loaded liposomal nanoparticles (LM-LNPs) against chlorfenapyr (CFP)-induced oxidative stress, antioxidant depletion, and DNA damage in renal tissue. (**A**) GSH (reduced glutathione), (**B**) SOD (superoxide dismutase), (**C**) CAT (catalase), (**D**) ROS (reactive oxygen species), (**E**) MDA (malondialdehyde), (**F**) PC (protein carbonyls), (**G**) NO (nitric oxide), and (**H**) DNA fragmentation. Data are presented as mean ± SEM (*n* = 7). Symbols denote significant differences (*p* < 0.05): * versus Control, † versus CFP, and ‡ versus CFP + LM.

The suppression of NRF2 signaling was accompanied by marked depletion of renal antioxidant defenses (Figure 5A–C). CFP reduced renal GSH content by approximately 50%, while SOD and CAT activities declined by approximately 65% and 52%, respectively, compared with controls. Treatment with LM partially restored these parameters, increasing GSH, SOD, and CAT by approximately 37%, 67%, and 44%, respectively, relative to the CFP group. The nano-liposomal formulation produced greater protection, increasing GSH by approximately 80%, SOD by 130%, and CAT by 70% compared with CFP-treated animals. Among these parameters, GSH levels in the LM-LNP group were restored to values approaching those observed in the control group.

Consistent with the compromised antioxidant status, CFP induced severe oxidative and nitrosative stress, as reflected by marked elevations in ROS, MDA, PC, and NO levels (Figure 5D–G). Relative to controls, CFP increased ROS by approximately 170%, MDA by 155%, PC by 190%, and NO by 245%. LM treatment attenuated these elevations, reducing ROS, MDA, PC, and NO by approximately 30%, 30%, 22%, and 10%, respectively, compared with the CFP group. In contrast, LM-LNPs achieved markedly greater reductions of approximately 53%, 52%, 39%, and 47%, respectively. Notably, MDA levels in the LM-LNP-treated group were restored to values close to those observed in control animals, indicating substantial attenuation of lipid peroxidation.

The oxidative damage induced by CFP was further evidenced by a greater than three-fold increase in renal DNA fragmentation compared with controls (Figure 5H). Treatment with LM reduced DNA fragmentation by approximately 24% relative to the CFP group, whereas LM-LNPs produced a more pronounced reduction of approximately 45%. Collectively, these findings demonstrate that CFP-induced suppression of NRF2 signaling resulted in antioxidant depletion, excessive oxidative stress, and DNA damage, while LM-LNPs provided significantly greater protection than free LM through restoration of redox homeostasis and enhancement of endogenous antioxidant defenses.

### 3.7. Effect of Limonene and LM-LNPs on Chlorfenapyr-Induced Activation of the NF-κB/COX-2 Inflammatory Pathway and Pro-Inflammatory Cytokines

**Figure 6:**
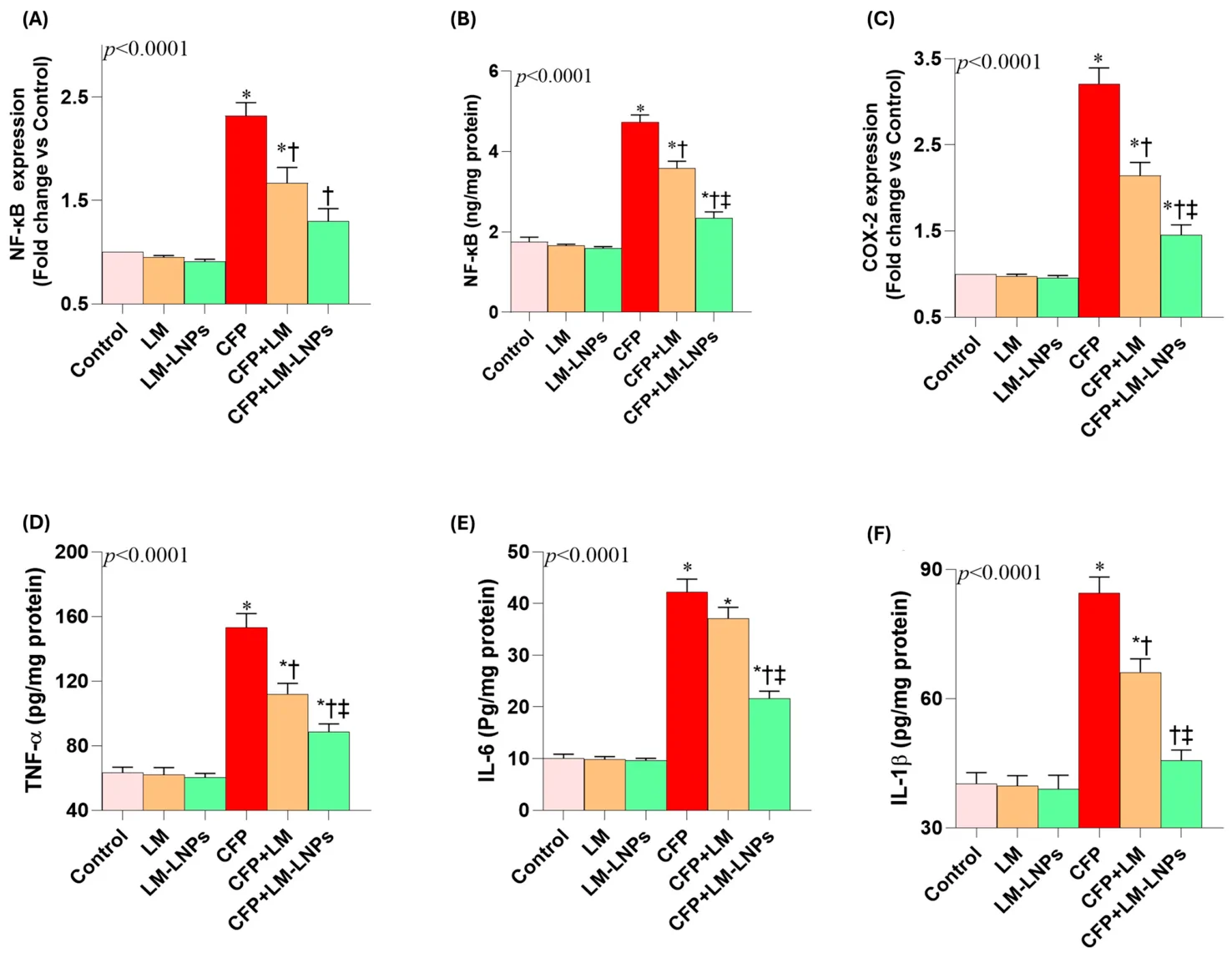
Protective effects of limonene (LM) and limonene-loaded liposomal nanoparticles (LM-LNPs) against chlorfenapyr (CFP)-induced activation of the NF-κB/COX-2 inflammatory pathway and pro-inflammatory cytokine production in renal tissue. (**A**) *NF-κB* (nuclear factor kappa B) gene expression, (**B**) NF-κB protein level, (**C**) *COX-2* (cyclooxygenase-2) gene expression, (**D**) TNF-α (tumor necrosis factor-alpha), (**E**) IL-6 (interleukin-6), and (**F**) IL-1β (interleukin-1 beta). Data are presented as mean ± SEM (*n* = 7). Symbols denote significant differences (*p* < 0.05): * versus Control, † versus CFP, and ‡ versus CFP + LM.

Chlorfenapyr (CFP) exposure induced a robust inflammatory response in renal tissue, as evidenced by significant activation of the NF-κB/COX-2 signaling pathway and increased production of pro-inflammatory cytokines (Figure 6A–F). Compared with the control group, CFP markedly activated inflammatory signaling, increasing *NF-κB* gene expression by approximately 2.3-fold, while NF-κB protein levels and *COX-2* expression increased by more than 3-fold, indicating robust activation of the NF-κB/COX-2 inflammatory pathway.

Co-treatment with limonene (LM) partially attenuated CFP-induced activation of the NF-κB/COX-2 pathway, reducing *NF-κB* gene expression, NF-κB protein levels, and COX-2 expression by approximately 29%, 24%, and 33%, respectively, relative to the CFP group. However, liposomal encapsulation markedly enhanced the anti-inflammatory efficacy of LM. LM-LNPs reduced *NF-κB* gene expression and protein levels by approximately 45% and decreased *COX-2* expression by 56%, indicating substantially greater suppression of inflammatory signaling than free LM. Notably, NF-κB gene expression in the LM-LNP-treated group approached control values, highlighting the ability of the nanoformulation to effectively counteract CFP-induced inflammatory activation.

Consistent with the activation of the NF-κB/COX-2 pathway, CFP elicited a robust inflammatory response characterized by approximately 140%, 320%, and 115% increases in renal TNF-α, IL-6, and IL-1β levels, respectively, compared with controls. LM treatment partially blunted these increases, whereas LM-LNPs produced a substantially greater anti-inflammatory effect, reducing all three cytokines by approximately 45–50% relative to the CFP group. Notably, IL-1β levels in LM-LNP-treated animals approached those observed in control animals. Together, these findings indicate that liposomal encapsulation significantly enhanced the anti-inflammatory efficacy of LM against CFP-induced renal injury.

### 3.8. Effect of Limonene and LM-LNPs on Chlorfenapyr-Induced Mitochondrial Dysfunction and Impaired Cellular Energy Metabolism

**Figure 7:**
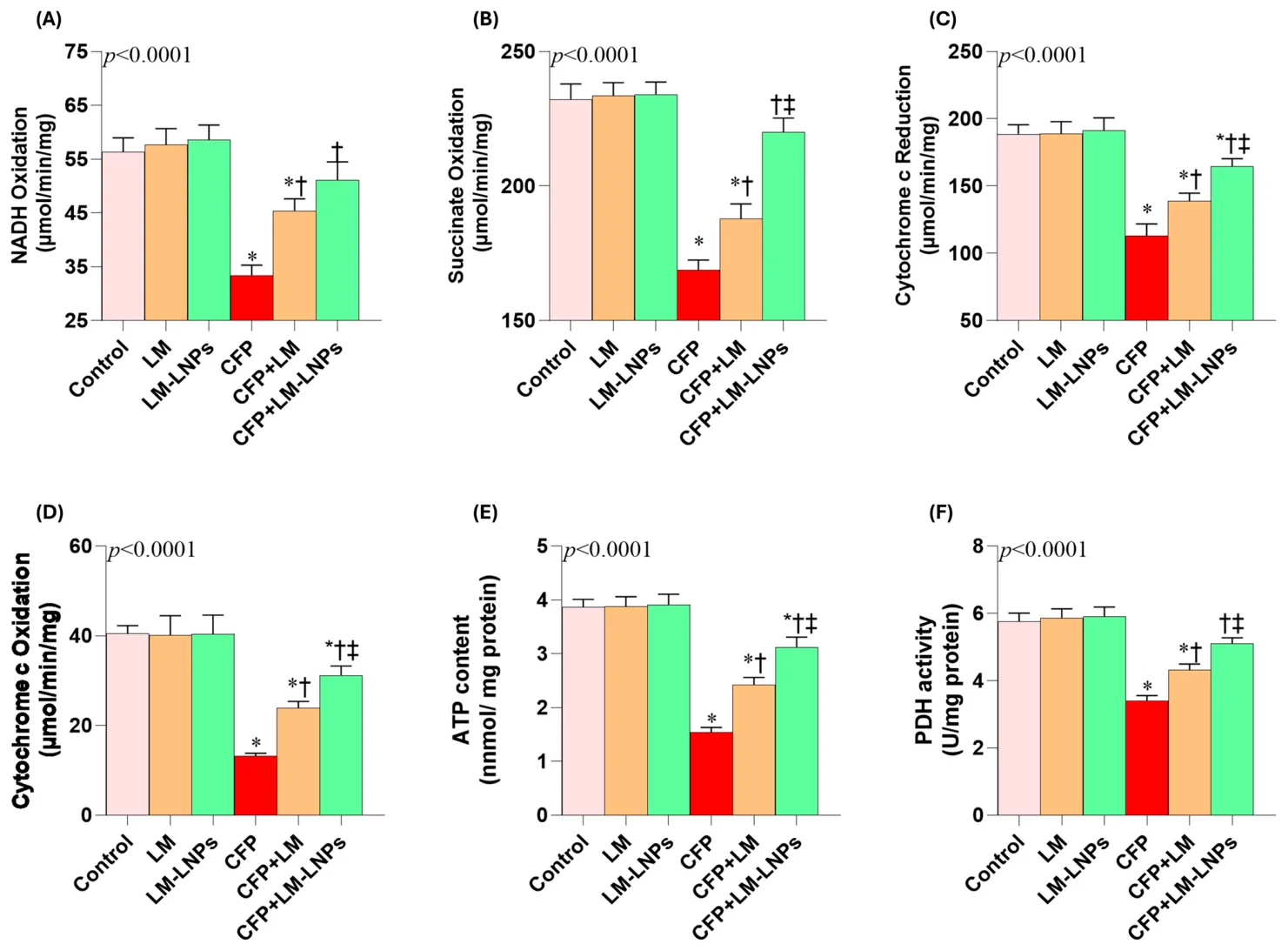
Protective effects of limonene (LM) and limonene-loaded liposomal nanoparticles (LM-LNPs) against chlorfenapyr (CFP)-induced mitochondrial dysfunction and impaired cellular energy metabolism in renal tissue. (**A**) NADH oxidation (nicotinamide adenine dinucleotide oxidation), (**B**) succinate oxidation, (**C**) cytochrome c reduction, (**D**) cytochrome c oxidation, (**E**) ATP (adenosine triphosphate) content, and (**F**) PDH (pyruvate dehydrogenase) activity. Data are presented as mean ± SEM (*n* = 7). Symbols denote significant differences (*p* < 0.05): * versus Control, † versus CFP, and ‡ versus CFP + LM.

Chlorfenapyr (CFP) exposure markedly impaired mitochondrial respiratory function, as evidenced by significant reductions in NADH oxidation, succinate oxidation, cytochrome c reduction, and cytochrome c oxidation compared with the control group (Figure 7A–D). Specifically, CFP reduced NADH oxidation by approximately 40%, succinate oxidation by 28%, cytochrome c reduction by 41%, and cytochrome c oxidation by 68%, indicating substantial disruption of mitochondrial electron transport chain activity. Co-treatment with limonene (LM) partially restored mitochondrial function, increasing these parameters by approximately 34%, 11%, 26%, and 83%, respectively, relative to the CFP group. The protective effect was markedly enhanced by liposomal encapsulation. Compared with CFP-treated animals, LM-LNPs increased NADH oxidation by approximately 53%, succinate oxidation by 29%, cytochrome c reduction by 47%, and cytochrome c oxidation by 138%. Notably, NADH and succinate oxidation in the LM-LNP-treated group approached values observed in control animals, indicating substantial recovery of mitochondrial respiratory activity.

The impairment of mitochondrial function was accompanied by marked disturbances in cellular energy metabolism (Figure 7E,F). CFP reduced renal ATP content and pyruvate dehydrogenase (PDH) activity by approximately 60% and 42%, respectively, compared with controls. LM treatment partially ameliorated these deficits, increasing ATP levels and PDH activity by approximately 57% and 27%, respectively, relative to the CFP group. In contrast, LM-LNPs produced a more pronounced restoration of cellular bioenergetics, increasing ATP content by approximately 105% and PDH activity by 51%. The improvement in PDH activity was particularly notable, with values approaching those observed in control animals. Collectively, these findings demonstrate that LM-LNPs more effectively preserved mitochondrial function and cellular energy homeostasis than free LM following CFP exposure.

### 3.9. Effect of Limonene and LM-LNPs on Chlorfenapyr-Induced Apoptotic Signaling in Renal Tissue

**Figure 8:**
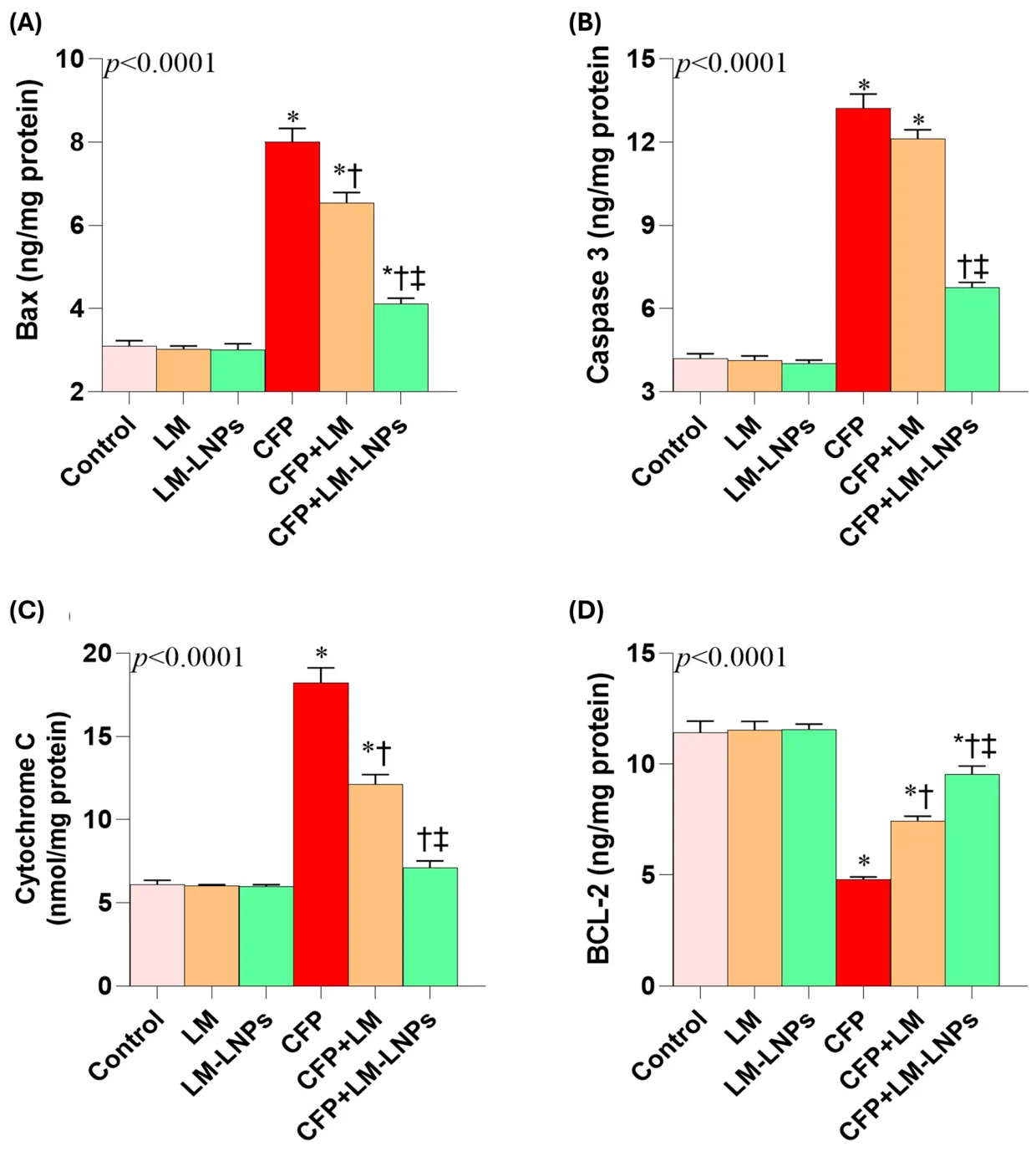
Protective effects of limonene (LM) and limonene-loaded liposomal nanoparticles (LM-LNPs) against chlorfenapyr (CFP)-induced mitochondrial-mediated apoptosis in renal tissue. (**A**) Bax (Bcl-2-associated X protein), (**B**) caspase-3 (cysteine-aspartate protease-3), (**C**) cytochrome c, and (**D**) Bcl-2 (B-cell lymphoma 2). Data are presented as mean ± SEM (*n* = 7). Symbols denote significant differences (*p* < 0.05): * versus Control, † versus CFP, and ‡ versus CFP + LM.

Consistent with the marked oxidative stress and mitochondrial dysfunction induced by CFP, renal apoptotic signaling was significantly activated, as evidenced by elevations in the pro-apoptotic markers Bax, caspase-3, and cytochrome c (Figure 8A–C). Compared with the control group, CFP increased Bax, caspase-3, and cytochrome c levels by approximately 2.6-fold, 3.2-fold, and 3.0-fold, respectively, indicating substantial activation of the mitochondrial apoptotic pathway.

Co-treatment with limonene (LM) partially attenuated these apoptotic alterations. Relative to the CFP group, LM reduced Bax, caspase-3, and cytochrome c levels by approximately 19%, 8%, and 33%, respectively. In contrast, LM-loaded liposomal nanoparticles (LM-LNPs) exerted a markedly stronger anti-apoptotic effect, reducing both Bax and caspase-3 levels by approximately 50% and cytochrome c levels by approximately 60% compared with CFP-treated animals. These findings indicate substantial suppression of mitochondrial-mediated apoptotic signaling by the nano-liposomal formulation.

Conversely, CFP markedly suppressed the anti-apoptotic protein Bcl-2 (Figure 8D), reducing its level by approximately 60% relative to controls. Both LM and LM-LNPs significantly restored Bcl-2 expression; however, the nano-liposomal formulation produced a substantially greater effect, increasing Bcl-2 levels by approximately two-fold relative to the CFP group, compared with a 58% increase observed with free LM. Collectively, these findings demonstrate that LM-LNPs more effectively attenuated CFP-induced mitochondrial apoptosis and promoted cell survival than non-encapsulated limonene.

### 3.10. Effect of Limonene and LM-LNPs on Chlorfenapyr-Induced Histopathological and Ultrastructural Renal Alterations

**Figure 9:**
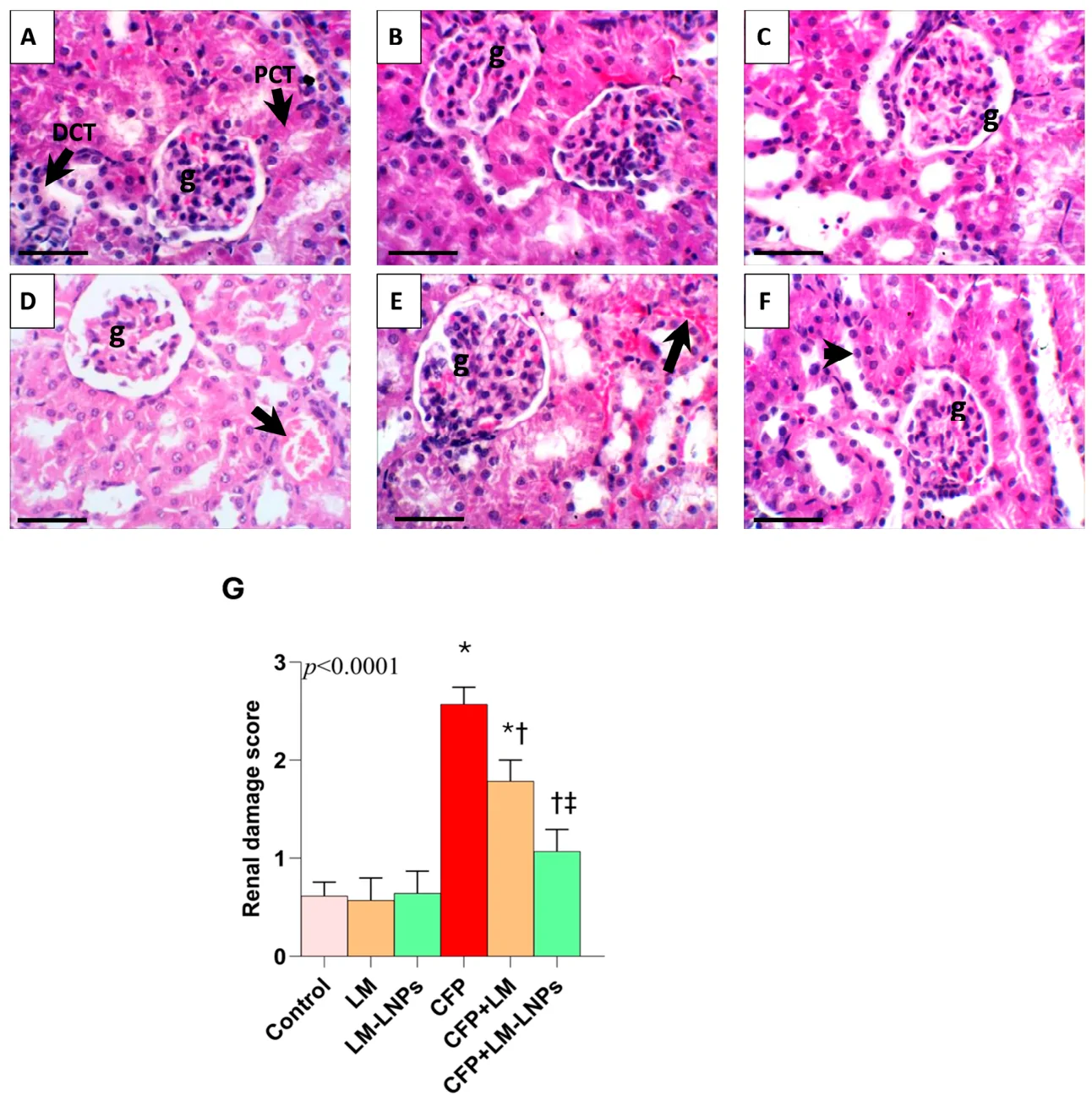
Representative photomicrographs of renal cortical sections illustrating the effects of limonene (LM) and limonene-loaded liposomal nanoparticles (LM-LNPs) on chlorfenapyr (CFP)-induced renal histopathological alterations. (**A**) Control; (**B**) LM; (**C**) LM-LNPs; (**D**) CFP; (**E**) CFP + LM; and (**F**) CFP + LM-LNPs groups. The labeled structures include proximal convoluted tubules (PCT), distal convoluted tubules (DCT), and glomeruli (g); arrows indicate vascular congestion. Although the representative images primarily depict the renal cortex, histopathological evaluation encompassed both the cortical and medullary regions. H&E staining; magnification, 400×; scale bar = 50 μm. (**G**) Semiquantitative renal histopathological injury scores. Three randomly selected animals were evaluated per group. For each animal, three kidney sections cut at different tissue levels were examined, with four randomly selected microscopic fields assessed per section, yielding 12 fields per animal. Field-level scores were averaged to obtain a single injury score for each animal. Data are presented as mean ± SEM (*n* = 3 animals per group). Symbols denote significant differences (*p* < 0.05): * versus Control, † versus CFP, and ‡ versus CFP + LM.

The control, LM, and LM-LNP groups exhibited preserved renal architecture across both the cortex and medulla (Figure 9A–C). Glomerular tufts remained intact within normal Bowman’s capsules, and the proximal and distal convoluted tubules retained their characteristic morphology without evidence of structural injury. The medullary region likewise showed normal tubular organization and no detectable histopathological abnormalities, indicating that neither free LM nor LM-LNPs produced discernible renal tissue damage.

In contrast, CFP exposure caused marked renal histopathological injury, most pronounced in the cortex (Figure 9D), including glomerular shrinkage, periglomerular inflammatory infiltration, and vascular congestion. The medulla was less affected, showing only mild tubular degeneration and vascular congestion. This pattern was reflected in the semiquantitative injury score, which was significantly higher than in controls (Figure 9G), confirming substantial CFP-induced nephrotoxicity.

Co-administration of LM partially alleviated these histopathological abnormalities, resulting in preservation of glomerular and tubular architecture and a reduction in renal injury scores (Figure 9E,G). The protective effect was more pronounced in the LM-LNP-treated group, which exhibited near-normal renal histology with minimal vascular congestion and well-preserved glomerular and tubular structures (Figure 9F). The mild medullary tubular degeneration and vascular congestion observed following CFP exposure were also less evident in both combination-treatment groups. Consistent with the overall histopathological findings, renal injury scores were reduced following treatment with LM and, to a greater extent, LM-LNPs (Figure 9G).

**Figure 10:**
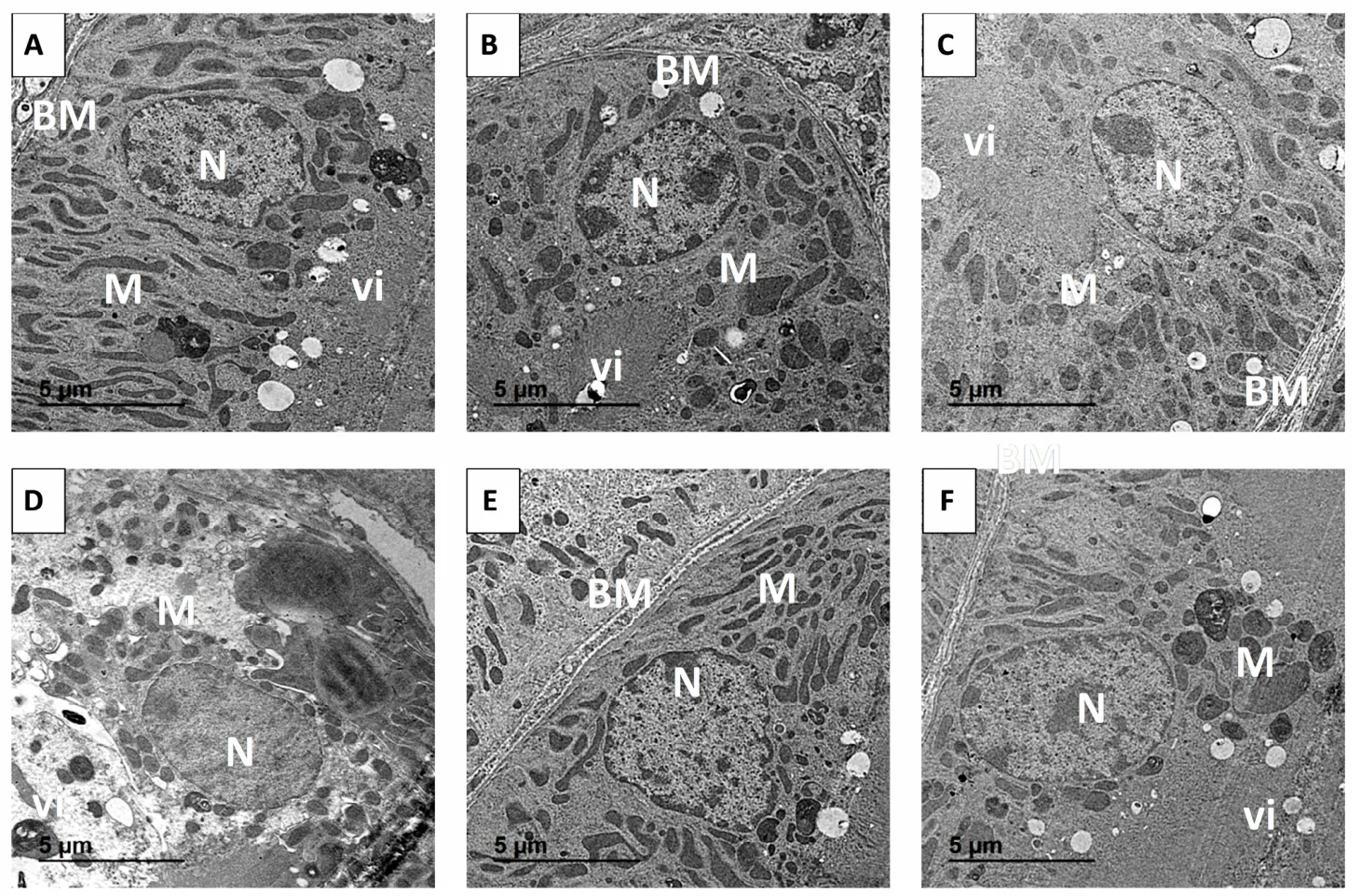
Representative transmission electron micrographs of renal cortex sections showing the protective effects of limonene (LM) and limonene-loaded liposomal nanoparticles (LM-LNPs) against chlorfenapyr (CFP)-induced ultrastructural renal damage. (**A**) Control group; (**B**) LM group; (**C**) LM-LNPs group; (**D**) CFP group; (**E**) CFP + LM group; and (**F**) CFP + LM-LNPs group. Cellular structures include nucleus (N), mitochondria (M), basement membrane (BM), and microvilli (vi). Scale bar = 5 μm.

Ultrastructural examination further supported the histopathological findings (Figure 10A–F). Renal cortical cells from the control, LM, and LM-LNP groups displayed normal ultrastructural features, including intact basement membranes, well-developed apical microvilli, euchromatic nuclei, and elongated mitochondria (Figure 10A–C). In contrast, CFP exposure resulted in pronounced ultrastructural alterations characterized by mitochondrial shrinkage and fragmentation, shortening and disruption of apical microvilli, and condensation of nuclear chromatin (Figure 10D), indicating severe cellular injury and mitochondrial damage.

Treatment with LM partially restored normal cellular ultrastructure, whereas LM-LNPs provided more substantial protection (Figure 10E,F). Renal cortical cells from LM-LNP-treated animals exhibited largely preserved mitochondrial morphology, intact microvilli, and euchromatic nuclei, closely resembling those observed in control tissues. These findings corroborate the biochemical and molecular data, demonstrating that liposomal encapsulation significantly enhanced the renoprotective efficacy of LM against CFP-induced renal injury.

### 3.11. Effect of Limonene and LM-LNPs on Renal NRF2 and NF-κB Immunoreactivity

**Figure 11:**
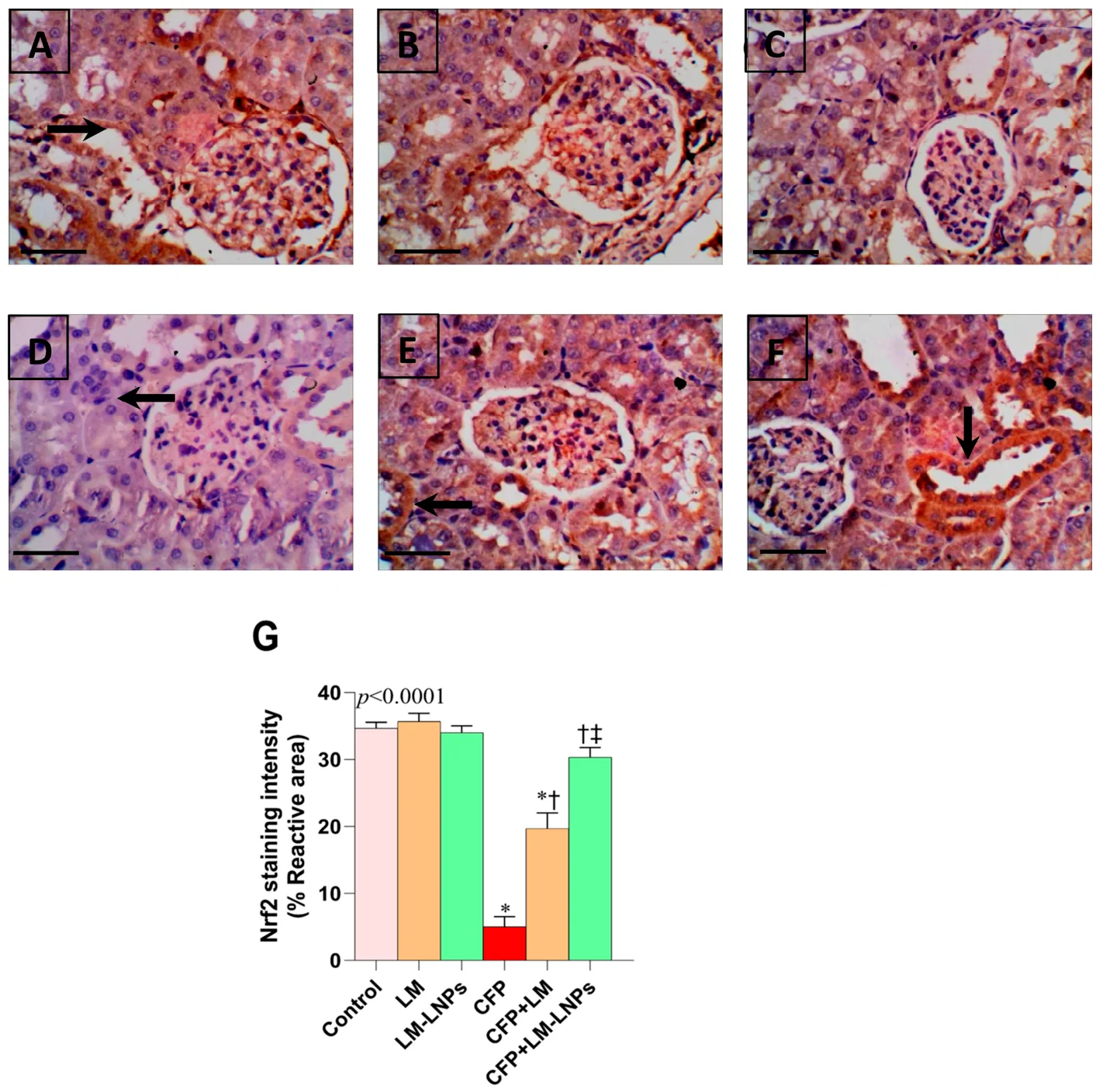
Protective effects of limonene (LM) and limonene-loaded liposomal nanoparticles (LM-LNPs) against chlorfenapyr (CFP)-induced suppression of NRF2 immunoreactivity in renal tissue. Representative photomicrographs of NRF2 immunostaining in kidney sections from (**A**) Control, (**B**) LM, (**C**) LM-LNPs, (**D**) CFP, (**E**) CFP + LM, and (**F**) CFP + LM-LNPs groups. Strong positive NRF2 immunoreactivity was observed in renal cortical tubular cells of the control, LM, and LM-LNPs groups, whereas CFP markedly reduced NRF2 staining intensity (arrows). Treatment with LM and LM-LNPs restored NRF2 immunoreactivity, with a more pronounced effect observed in the LM-LNP-treated group. Original magnification: 400×; scale bar = 50 μm. (**G**) Quantitative analysis of NRF2 immunostaining intensity (% reactive area). Immunohistochemical analysis was performed on three randomly selected animals per group. For each animal, three kidney sections cut at different tissue levels were examined, and five randomly selected microscopic fields per section were evaluated (15 fields per animal). Data are presented as mean ± SEM (*n* = 3 animals per group). Symbols denote significant differences (*p* < 0.05): * versus Control, † versus CFP, and ‡ versus CFP + LM.

**Figure 12:**
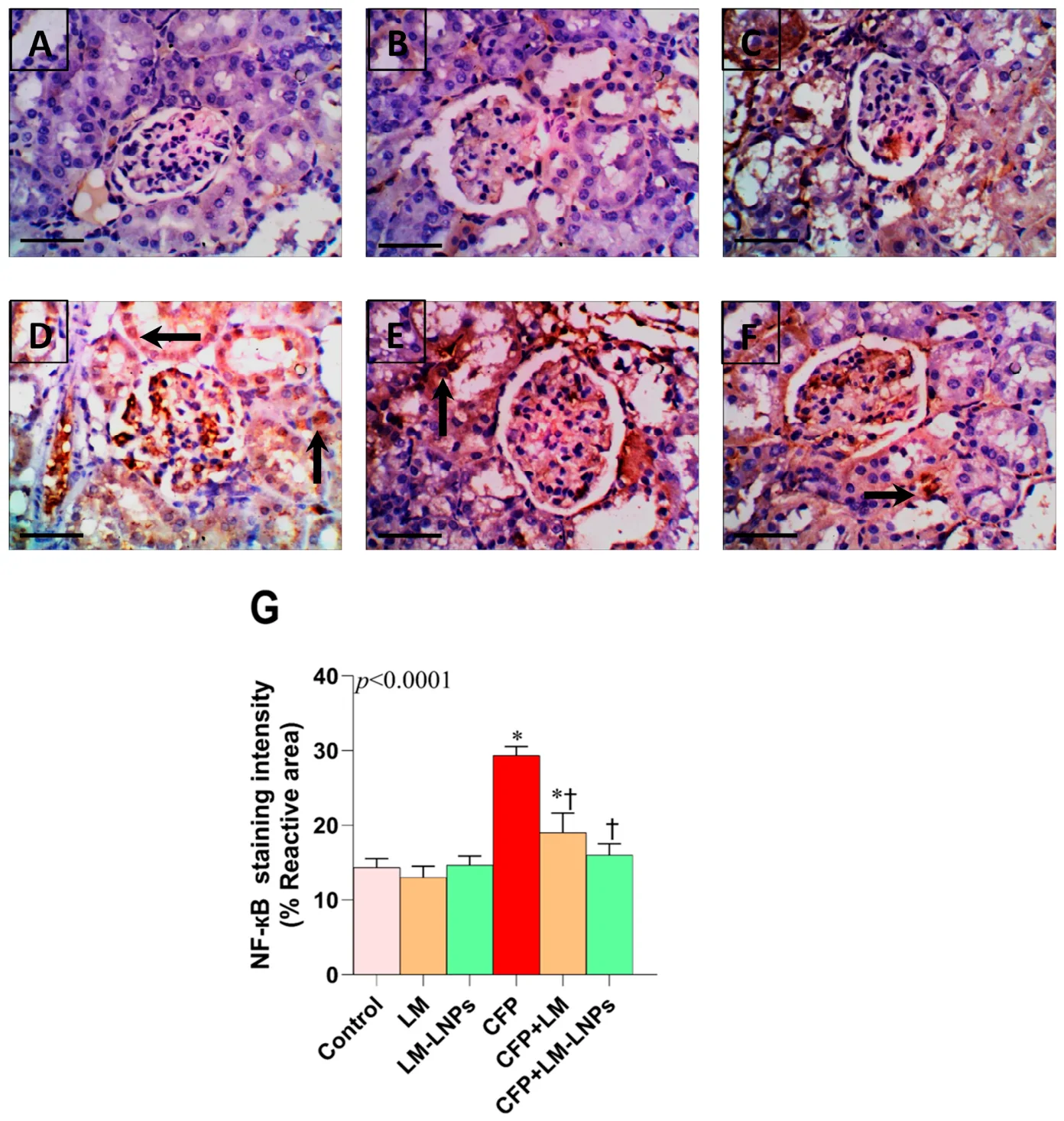
Protective effects of limonene (LM) and limonene-loaded liposomal nanoparticles (LM-LNPs) against chlorfenapyr (CFP)-induced NF-κB activation in renal tissue. Representative photomicrographs of NF-κB immunostaining in kidney sections from (**A**) Control, (**B**) LM, (**C**) LM-LNPs, (**D**) CFP, (**E**) CFP + LM, and (**F**) CFP + LM-LNPs groups. Minimal basal NF-κB immunoreactivity was observed in the control, LM, and LM-LNPs groups, whereas CFP markedly increased NF-κB expression, as evidenced by intense positive staining in renal tubular epithelial cells (arrows). Treatment with LM and LM-LNPs attenuated NF-κB immunoreactivity, with a more pronounced reduction observed in the LM-LNP-treated group. Original magnification: 400×; scale bar = 50 μm. (**G**) Quantitative analysis of NF-κB immunostaining intensity (% reactive area). Histopathological analysis was performed on three randomly selected animals per group. For each animal, three kidney sections cut at different tissue levels were examined, and five randomly selected microscopic fields per section were evaluated (15 fields per animal). Data are presented as mean ± SEM (*n* = 3 animals per group). Symbols denote significant differences (*p* < 0.05): * versus Control, and † versus CFP.

Immunohistochemical analysis revealed reciprocal alterations in renal NRF2 and NF-κB expression following CFP exposure (Figure 11 and Figure 12). In the control, LM, and LM-LNPs groups, strong NRF2 immunoreactivity was observed in renal cortical tubular cells (Figure 11A–C), indicating intact antioxidant defense mechanisms. In contrast, CFP markedly reduced NRF2 staining intensity (Figure 11D), consistent with suppression of the NRF2 antioxidant pathway. Quantitative analysis demonstrated an approximately 85% reduction in NRF2 immunoreactivity compared with the control group (Figure 11G). Co-treatment with LM partially restored NRF2 expression, whereas LM-LNPs produced a substantially greater effect, increasing NRF2 immunoreactivity by approximately 60% relative to the CFP group and restoring staining intensity toward control levels.

An opposite pattern was observed for NF-κB immunoreactivity (Figure 12A–G). Renal tissues from the control, LM, and LM-LNPs groups exhibited only minimal basal NF-κB staining (Figure 12A–C). However, CFP exposure induced marked NF-κB activation, evidenced by intense positive immunostaining in renal tubular epithelial cells (Figure 12D). Quantitative analysis showed an approximately two-fold increase in NF-κB immunoreactivity compared with controls (Figure 12G), confirming activation of inflammatory signaling pathways. Treatment with LM attenuated this response, while LM-LNPs exerted a more pronounced inhibitory effect, reducing NF-κB staining intensity by approximately 45% relative to the CFP group. Consequently, NF-κB immunoreactivity in the LM-LNP-treated group approached levels observed in control animals.

Collectively, these findings provide histological evidence that CFP disrupts the balance between antioxidant and inflammatory signaling by suppressing NRF2 and activating NF-κB. Moreover, the superior restoration of NRF2 expression and suppression of NF-κB immunoreactivity observed with LM-LNPs further supports the enhanced renoprotective efficacy of the nano-liposomal formulation compared with free LM.

## 4. Discussion

This study provides a multilevel mechanistic characterization of CFP-induced renal toxicity and a comparative evaluation of the protective efficacy of limonene (LM) and its nanoliposomal formulation (LM-LNPs). By integrating biochemical, molecular, histopathological, ultrastructural, and immunohistochemical endpoints, the study establishes a coherent link between functional impairment and the underlying signaling pathways involved in renal injury. The findings suggest that CFP-induced nephrotoxicity is not a singular event but rather a cascade initiated by mitochondrial dysfunction, amplified by oxidative stress, and propagated through inflammatory and apoptotic signaling networks.

CFP exposure resulted in marked elevations in serum urea, creatinine, and uric acid, indicating compromised glomerular filtration and tubular dysfunction, consistent with impaired renal clearance mechanisms [34,35]. Rather than representing isolated functional abnormalities, these changes likely reflect upstream mitochondrial and redox disturbances that disrupt energy-dependent transport processes in renal tubular cells. Treatment with LM significantly improved these parameters, whereas LM-LNPs produced a greater degree of protection, suggesting that liposomal encapsulation enhanced the biological activity of LM. The superior efficacy of LM-LNPs may be attributed to improved stability, bioavailability, and cellular delivery of LM, facilitating more effective interaction with intracellular targets and greater tissue retention.

Mitochondrial dysfunction appears to be a central event in CFP-induced nephrotoxicity. This was demonstrated by a marked decline in the activities of mitochondrial respiratory complexes I-IV. The observed reductions in mitochondrial complex activities indicate impaired electron transport chain function and compromised oxidative phosphorylation [36]. Such mitochondrial impairment can reduce ATP generation while promoting excessive reactive oxygen species (ROS) production and disruption of cellular redox homeostasis [37]. Consistent with this mechanism, CFP exposure increased ROS and NO levels, together with elevated MDA and protein carbonyl content, indicating lipid peroxidation and protein oxidation, respectively, which are hallmarks of oxidative macromolecular damage. Concurrent depletion of GSH, SOD, and CAT further reflects the collapse of endogenous antioxidant defenses under sustained oxidative stress [38]. In addition, altered PDH activity suggests impaired metabolic adaptation and disrupted cellular energy metabolism, contributing to ATP depletion, ROS overproduction, and activation of apoptotic pathways [1,39]. Notably, liposomal encapsulation enhanced the ability of LM to preserve mitochondrial bioenergetics, as reflected by greater restoration of ATP levels and respiratory chain enzyme activities.

Importantly, CFP markedly suppressed NRF2 and its downstream targets HO-1 and NQO1, indicating inhibition of the principal cytoprotective transcriptional axis responsible for cellular redox adaptation [40]. Immunohistochemical findings corroborated these results, demonstrating reduced NRF2 immunoreactivity in renal tissues, which mechanistically explains the diminished expression of antioxidant genes [41]. The inhibitory effect of CFP on the NRF2/HO-1 pathway is consistent with previous studies describing CFP-induced oxidative injury in hepatic and hippocampal tissues [42,43]. Conversely, LM treatment restored NRF2 signaling, whereas LM-LNPs produced a greater recovery of the NRF2/HO-1 axis, suggesting that liposomal encapsulation enhanced intracellular availability of LM and strengthened activation of endogenous antioxidant defenses. This restoration likely underlies the observed normalization of oxidative stress biomarkers and preservation of cellular integrity. These findings are consistent with previous studies highlighting NRF2 activation by LM and the therapeutic advantages of nano-delivery systems [16,17]. Furthermore, the marked increase in DNA fragmentation observed following CFP exposure reflects ROS-mediated genotoxic stress [44], which was significantly attenuated by LM, particularly in its nanoliposomal formulation, indicating effective protection against oxidative DNA damage and preservation of genomic stability.

Inflammation represents a secondary but closely interconnected response to oxidative stress, primarily mediated through NF-κB activation. CFP exposure upregulated NF-κB and its downstream effectors, including TNF-α, IL-1β, IL-6, and COX-2, indicating activation of a pro-inflammatory transcriptional program that contributes to renal injury [45,46]. The interplay between ROS and NF-κB signaling likely establishes a self-amplifying cycle that sustains inflammation and accelerates tissue damage [47]. This mechanism was further supported by strong NF-κB immunoreactivity in renal tissues following CFP exposure. LM treatment attenuated activation of this pathway, while LM-LNPs produced a greater reduction in inflammatory mediators, supporting the close mechanistic relationship between oxidative stress and NF-κB-driven inflammation.

Apoptosis represents a major downstream consequence of persistent oxidative stress and mitochondrial dysfunction [48,49]. CFP exposure increased the BAX/BCL-2 ratio, indicating mitochondrial membrane destabilization and activation of intrinsic apoptotic signaling. This was accompanied by cytochrome c release and caspase-3 activation, confirming execution of the apoptotic program [50,51]. These events are mechanistically linked to mitochondrial permeability transition and ROS-induced cellular damage [52]. LM treatment mitigated these alterations, whereas LM-LNPs provided greater protection against apoptotic signaling, likely through improved preservation of mitochondrial integrity and more effective suppression of upstream oxidative stress.

The biochemical and molecular findings were strongly corroborated by histopathological and ultrastructural observations, providing structural validation of functional recovery. CFP-induced renal injury was characterized by glomerular distortion, tubular degeneration, inflammatory cell infiltration, vascular alterations, mitochondrial swelling, and cristae disruption, consistent with previous reports describing renal damage associated with oxidative stress, inflammation, and apoptosis [13,53]. Treatment with LM, particularly in its nano-liposomal formulation, markedly improved renal architecture and preserved mitochondrial integrity, including normalization of cristae morphology and organelle structure, in agreement with previous studies demonstrating the enhanced antioxidant and cytoprotective properties of nano-encapsulated limonene [54]. These structural findings provide morphological confirmation of the biochemical and molecular improvements observed following LM-LNP treatment.

The enhanced renoprotective efficacy of LM-LNPs is consistent with previous reports demonstrating that lipid-based nanoformulations improve the oral delivery of limonene and other lipophilic terpenes. Nanoencapsulation protects limonene from volatilization and degradation while enhancing its aqueous dispersibility, gastrointestinal absorption, systemic bioavailability, and tissue distribution [55,56]. Koch et al. reported that a self-microemulsifying drug delivery system increased the oral bioavailability of limonene by approximately 3.7-fold and substantially enhanced its accumulation in multiple organs, including the kidney [57]. Furthermore, recent reviews have demonstrated that nanoencapsulation improves the pharmacokinetic properties and therapeutic efficacy of limonene and other terpene-based compounds by enhancing their stability, prolonging drug release, and increasing tissue exposure [55,56]. Collectively, these findings provide a plausible explanation for the greater biological activity observed with LM-LNPs in the present study.

In summary, CFP-induced nephrotoxicity appears to be driven by a mitochondria-centered mechanism involving oxidative stress, inflammatory amplification, and apoptotic cell death. LM exerts renoprotective effects through modulation of these interconnected pathways, while nano-liposomal delivery enhances its biological activity and strengthens its ability to restore mitochondrial function, redox balance, and cellular homeostasis.

The present findings should be interpreted in light of several study limitations. First, the study was conducted in a single animal model, and validation in additional experimental models is warranted before extrapolating these findings to human exposure scenarios. Second, although chlorfenapyr and limonene were administered sequentially to minimize the possibility of direct physicochemical interaction within the gastrointestinal tract, their chemical compatibility was not directly evaluated. Therefore, the possibility of limited physicochemical interactions cannot be completely excluded. Third, pharmacokinetic profiling, plasma concentration measurements, and renal tissue distribution analyses of LM and LM-LNPs were not performed, precluding direct confirmation of the enhanced bioavailability and renal accumulation of the nanoformulation. Nevertheless, the greater efficacy of LM-LNPs observed in the present study is consistent with previous reports demonstrating improved oral bioavailability and tissue distribution of lipid-based limonene nanoformulations. In addition, encapsulation efficiency and the in vitro release profile were determined using UV spectrophotometry. Although appropriate blank liposomal formulations were used to minimize background interference during quantification, chromatographic techniques such as GC-MS or GC-FID would provide greater analytical specificity for limonene quantification and should be considered in future studies. Fourth, histopathological and ultrastructural analyses were performed on three randomly selected animals per group. Although the histopathological findings were consistent with the biochemical and molecular results, future studies incorporating a larger number of biological replicates would further strengthen the histopathological assessment and better capture biological variability. In addition, the mechanistic investigation focused primarily on oxidative stress, inflammation, apoptosis, and the NRF2/HO-1 and NF-κB/COX-2 signaling pathways. Future studies should further investigate additional mechanisms potentially involved in the renoprotective effects of LM-LNPs, including autophagy, endoplasmic reticulum stress, epigenetic regulation, inflammatory cell infiltration, and mitochondrial homeostasis, as well as biomarkers such as CD45, CXCL1, CD68, CD32, PGC1α, p62, VDAC, and ATP synthase. Finally, long-term safety, dose–response relationships, complementary in vitro validation using renal cell lines, and quantitative digital morphometric analyses of renal lesions were beyond the scope of the present study. Future studies incorporating these approaches will further strengthen the mechanistic understanding and translational potential of LM-LNPs for the prevention of CFP-induced renal injury.

## 5. Conclusions

In conclusion, chlorfenapyr-induced nephrotoxicity is characterized by a complex interplay of mitochondrial dysfunction, oxidative stress, inflammation, and apoptosis, culminating in significant functional, molecular, histopathological, and ultrastructural renal damage. Limonene provided substantial protection against these toxic effects through modulation of redox homeostasis, inflammatory signaling, and mitochondrial integrity. Importantly, nano-liposomal encapsulation markedly enhanced the renoprotective efficacy of limonene, resulting in greater restoration of antioxidant defenses, activation of the NRF2 pathway, suppression of NF-κB-mediated inflammation, preservation of mitochondrial function, and improvement of renal architecture. Collectively, these findings highlight the therapeutic potential of nano-liposomal limonene as a promising strategy for mitigating chlorfenapyr-induced renal injury and support further investigation of this nanoformulation in translational models of toxicant-induced nephrotoxicity.

## Data Availability

The data presented in this study are available on request from the corresponding author.

## Supplementary Materials

The following supporting information can be downloaded at: https://www.mdpi.com/article/10.3390/toxics14080668/s1, Table S1: Commercial assay kits, reagents, and analytical systems used for the determination of serum biochemical parameters, oxidative stress biomarkers, inflammatory mediators, apoptosis-related proteins, mitochondrial function, DNA fragmentation, and RT-qPCR analyses; Table S2: Primer sequences used for quantitative real-time PCR (RT-qPCR) analysis of target genes; Table S3: Semi-Quantitative Assessment of Renal Histopathological Alterations; Methods S1: Preparation of Limonene -loaded liposomal nanoparticles (LM-LNPs); Methods S2: Determination of Encapsulation Efficiency and Drug Loading in LM-LNPs; Methods S3: In Vitro Release Profile of Limonene from LM-LNPs; Methods S4: Fourier Transform Infrared (FTIR) Spectroscopic Characterization of LM-LNPs; Methods S5: Histopathological and Ultrastructural Examination; Methods S6: RNA Isolation and RT-qPCR Analysis; Methods S7: Assessment of Mitochondrial Respiratory Chain Complex Activities; Methods S8: Immunohistochemical Assay of NRF2 and NF-κB.
