# Supplementary material for: Protective Effects of Limonene and a Nano-Liposomal Limonene Formulation Against Chlorfenapyr-Induced Renal Toxicity: Mechanistic Insights into NRF2/HO-1 and NF-κB/COX-2 Signaling, Mitochondrial Dysfunction, and Apoptosis in Rat Kidneys
Source: Toxics. 2026 Jul 28;14(8):668. doi: 10.3390/toxics14080668 (PMC13517275; doi:10.3390/toxics14080668)
Supplement: Supplementary file 1 [file toxics-14-00668-s001.zip › toxics-4399168-supplementary.pdf]

Supplementary Tables (S1-S3)

Table S1. Commercial assay kits, reagents, and analytical systems used for the determination of serum biochemical parameters, oxidative stress biomarkers, inflammatory mediators, apoptosis-related proteins, mitochondrial function, DNA fragmentation, and RT-qPCR analyses. All assays were performed according to the manufacturers' instructions. Detailed experimental procedures are provided in the Supplementary Methods.

Assay/Analyte	Assay Principle	Manufacturer	Catalog No.
Serum creatinine	Colorimetric assay	Diagnostic, Giza, Egypt	CR-12-50
Serum urea	Colorimetric assay	Diagnostic, Giza, Egypt	UR-21-10
Serum uric acid	Colorimetric assay	Diagnostic, Giza, Egypt	UA-21-20
GSH)	Colorimetric assay	BioDiagnostic, Giza, Egypt	GR 2511
SOD	Colorimetric assay	BioDiagnostic, Giza, Egypt	SOD 2521
CAT	Colorimetric assay	BioDiagnostic, Giza, Egypt	CA 2517
MDA	TBARS colorimetric assay	BioDiagnostic, Giza, Egypt	MD 2529
PC	ELISA	MyBioSource, San Diego, CA, USA	MBS760520
ROS	Fluorescence assay (DCFH-DA)	MyBioSource, San Diego, CA, USA	MBS2540517
NRF2	Sandwich ELISA	Abcam, Cambridge, UK	ab207223
HO-1	Sandwich ELISA	Abcam, Cambridge, UK	ab279414
Caspase-3	ELISA	MyBioSource, San Diego, CA, USA	MBS261814
Bax	ELISA	MyBioSource, San Diego, CA, USA	MBS2512405
Bcl-2	ELISA	MyBioSource, San Diego, CA, USA	MBS704498
Cytochrome *c*	ELISA	Elabscience Biotechnology Inc., Houston, TX, USA	E-EL-R0006
TNF-α	ELISA	Assay Genie, Dublin, Ireland	AEES00516
IL-6	ELISA	Assay Genie, Dublin, Ireland	RTFI00034
IL-1β	ELISA	Assay Genie, Dublin, Ireland	RTES00294
NF-κB p65	SimpleStep ELISA	Abcam, Cambridge, UK	ab176648
DNA fragmentation	ELISA	MyBioSource, San Diego, CA, USA	MBS270898
RNA extraction	QIAzol Lysis Reagent	Qiagen, Hilden, Germany	79306*
cDNA synthesis	iScript™ cDNA Synthesis Kit	Bio-Rad, Hercules, CA, USA	1708891*
RT-qPCR Master Mix	iTaq™ Universal SYBR® Green Supermix	Bio-Rad, Hercules, CA, USA	1725121

Assay/Analyte	Assay Principle	Manufacturer	Catalog No.
ATP	ATP Assay Kit	Jiancheng Bioengineering Institute, Nanjing, China	-
PDH	Enzyme activity assay	Solarbio, Beijing, China	-

Abbreviations: ATP, adenosine triphosphate; Bax, Bcl-2-associated X protein; Bcl-2, B-cell lymphoma 2 protein; CAT, catalase; GSH, reduced glutathione; HO-1, heme oxygenase-1; IL, interleukin; MDA, malondialdehyde; NF-κB, nuclear factor kappa B; NRF2, nuclear factor erythroid 2-related factor 2; PC, protein carbonyl; PDH, pyruvate dehydrogenase; ROS, reactive oxygen species; SOD, superoxide dismutase; TBARS, thiobarbituric acid reactive substances; TNF-α, tumor necrosis factor-α.

Table S2. Primer sequences used for the quantitative real-time PCR (RT-qPCR) analysis of target genes.

Gene	Sequences (5′-3′)	Accession no	Length (bp)
Nrf2	F: TTGTAGATGACCATGAGTC<br>R: TGTCCTGCTGTATGCTGCTT	NM_031789.2	141
HO-1	F: ATGTCCCAGGATTTGTCCGA<br>R: ATGGTACAAGGAGGCCATCA	NM_012580.2	144
NQO1	F: CTGGCCAATTCAGAGTGGCA<br>R: GATCTGGTTGTCGGCTGGAA	NM_017000.3	304
NF-κB	F: AGTCCCGCCCCTTCTAAAAC<br>R: CAATGGCCTCTGTGTAGCCC	NM_001276711.1	106
Cox-2	F: CTCCTTGAACACGGACTTGC<br>R: TCAGGGAGAAGCGTTTGC	NM_017232.3	150
β-Actin	F: CAGCCTTCCTTCTTGGGTATG<br>R: AGCTCAGTAACAGTCCGCCT	NM_031144.3	360

Abbreviations: Nrf2, nuclear factor erythroid 2-related factor 2; HO-1, heme oxygenase-1; NQO1, NAD(P)H quinone dehydrogenase 1; NF-κB, nuclear factor kappa B; COX-2, cyclooxygenase-2; β-actin, beta-actin.

Table S3. Semi-quantitative assessment of renal histopathological alterations.

Score	Renal tubular injury and necrotic changes in tissue sections	Alterations in glomerular architecture	Inflammation	Hemorrhage
**0**	None	None	None	None
**1**	Focal degenerative alterations were identified in 1-2 of the 12 observed kidney fields.	Glomerular atrophy of mild degree was identified in 1-2 kidney sections.	Kidney sections showed mild inflammation in 1-2 of the examined fields.	Kidney sections showed mild congestion with sporadic hemorrhagic spots in 1-2 samples.
**2**	Among the 12 kidney fields evaluated, 3-6 exhibited degeneration	In 3-4 of the 12 evaluated kidney fields, mild to	In 3-4 of the kidney sections evaluated, moderate	Kidney sections showed mild interstitial congestion

	along with focal epithelial desquamation.	moderate glomerular atrophy was detected.	inflammation was detected.	and focal bleeding in 3-4 samples.
**3**	In 7-9 of the 12 examined kidney fields, severe tubular injury was evident.	In 5-6 of the kidney fields evaluated, lamellar layers appeared fused.	Among the kidney fields assessed, 5-7 exhibited severe inflammatory cell infiltration.	In 5-6 kidney fields, marked vascular congestion and focal bleeding were identified.

Supplementary Methods (S1-S8)

Method S1. Preparation of Limonene -loaded liposomal nanoparticles (LM-LNPs)

Limonene, cholesterol, and L-α-phosphatidylcholine were purchased from Sigma-Aldrich (St. Louis, MO, USA). Limonene-loaded liposomal nanoparticles (LM-LNPs) were prepared using the thin-film hydration method, as previously described [28]. Briefly, 70 mg of L-α-phosphatidylcholine (0.09 mmol), 30 mg of cholesterol (0.078 mmol), and 1 mg of limonene (5 μmol) were dissolved in 10 mL of chloroform/methanol (2:1, v/v) in a round-bottom flask. The organic solvent was removed under reduced pressure to form a uniform thin lipid film. The resulting film was hydrated with 5 mL of phosphate-buffered saline (PBS; pH 7.4) to produce multilamellar vesicles.

The vesicle suspension was sonicated using a SONICS Vibra-Cell ultrasonic processor at 30% amplitude with alternating 10 s on/off cycles for 10 min to obtain a well-dispersed nanoscale formulation. To minimize thermal degradation, sonication was performed on ice. Following hydration, the liposomal suspension was gently agitated at 37 °C for 30 min to promote uniform limonene incorporation. The final formulation contained limonene at a concentration of 200 μg/mL. Unencapsulated limonene was removed by centrifugation at 15,000 × g for 30 min to obtain purified LM-LNPs.

Physicochemical characterization of LM-LNPs included the determination of particle size (Z-average), the polydispersity index (PDI), and zeta potential. Particle size measurements were performed in distilled water, whereas zeta potential measurements were conducted in triplicate to evaluate colloidal stability and surface charge. The morphology and ultrastructural characteristics of LM-LNPs were examined using a JEOL JEM-2100 high-resolution transmission electron microscope (JEOL Ltd., Tokyo, Japan) operated at 160 kV.

Method S2. Determination of Encapsulation Efficiency and Drug Loading in LM-LNPs

The encapsulation efficiency of limonene-loaded liposomal nanoparticles (LM-LNPs) was determined by measuring the amount of unencapsulated limonene remaining in the supernatant following centrifugation. Free limonene concentration was quantified by UV-Vis spectrophotometry at 210 nm using a calibration curve over the concentration range of 2–20 μg/mL. Blank liposomal formulations containing all lipid components but no limonene were analyzed under identical conditions to account for background absorbance arising from phosphatidylcholine and cholesterol. The amount of encapsulated limonene was calculated by subtracting the amount of free limonene detected in the supernatant from the total amount of limonene initially added. Drug loading (DL) and encapsulation efficiency (EE) were subsequently calculated using the below equations. Drug loading was expressed as the percentage of encapsulated limonene relative to the total mass of the liposomal formulation, whereas encapsulation efficiency was expressed as the percentage of the initial limonene successfully entrapped within the liposomes:

EE (%) = [(Total limonene - Free limonene) / Total limonene] × 100

DL (%) = [(Total limonene - Free limonene) / Total mass of liposomal formulation] × 100

Method S3. In Vitro Release Profile of Limonene from LM-LNPs

The in vitro release profile of limonene from LM-LNPs was evaluated using the dialysis bag diffusion method and compared with that of free limonene. To maintain sink conditions, an aliquot of the LM-LNP formulation was placed in a dialysis membrane (molecular weight cut-off [MWCO]: 12–14 kDa) and immersed in 50 mL of phosphate-buffered saline (PBS, pH 7.4) containing 0.5% Tween 80. The release study was performed at 37 °C under constant agitation. At predetermined time intervals, aliquots of the release medium were withdrawn and replaced with an equal volume of fresh release medium to maintain a constant volume and sink conditions. The amount of released limonene was quantified by UV-Vis spectrophotometry at 210 nm using a calibration curve, with PBS containing 0.5% Tween 80 serving as the blank. The cumulative percentage of limonene released was calculated and plotted as a function of time.

Method S4. Fourier Transform Infrared (FTIR) Spectroscopic Characterization of LM-LNPs

FTIR spectroscopy was employed to investigate potential interactions between limonene and the lipid components of LM-LNPs and to confirm successful drug encapsulation. Lyophilized samples of pure limonene, blank liposomes, and LM-LNPs were analyzed over a spectral range of 4000-400 cm^{-1}. The resulting spectra were examined for characteristic functional group vibrations and for shifts in peak position, changes in intensity, or peak broadening that could indicate intermolecular interactions between limonene and the liposomal matrix. These analyses were used to assess the structural integrity of the formulation and verify the incorporation of limonene within the lipid bilayer.

Method S5. RNA Isolation and RT-qPCR Analysis

Total RNA was extracted from kidney tissues utilizing QIAzol Lysis Reagent (Qiagen, Germany) in accordance with the manufacturer's guidelines. Tissue samples were homogenized with a TissueLyser II homogenizer, followed by phase separation using chloroform and centrifugation at 12,000 × g for 15 minutes. The aqueous phase was collected, and RNA was precipitated using isopropanol, followed by washing with 75% ethanol and resuspension in nuclease-free water. RNA concentration and purity were evaluated spectrophotometrically before cDNA synthesis. Complementary DNA (cDNA) was synthesized from purified RNA utilizing the iS-cript™ cDNA Synthesis Kit (Bio-Rad, Hercules, CA, USA) in accordance with the manufacturer's protocol. Quantitative real-time PCR (RT-qPCR) was conducted utilizing iTaq™ Universal SYBR® Green Supermix (Bio-Rad, Cat. No. 172-5121) on a Rotor-Gene Q Real-Time PCR System (Qiagen, Germany). Gene-specific primers were sourced from Macrogen in Seoul, South Korea (Table S2). Relative mRNA expression levels were assessed utilizing the comparative 2-ΔΔCt method, as described by Livak and Schmittgen [33]. Gene expression was normalized to the housekeeping gene and presented as fold change compared to the control group.

Method S6. Assessment of Mitochondrial Respiratory Chain Complex Activities

The activities of mitochondrial electron transport chain (ETC) complexes I-IV were determined spectrophotometrically in kidney homogenates using established methods. Complex I (NADH dehydrogenase) activity was measured by monitoring NADH oxidation at 340 nm according to the method of Galante and Hatefi [34]. Complex II (succinate dehydrogenase) activity was assessed by measuring the

reduction of 2,6-dichlorophenolindophenol (DCIP) at 600 nm, as described by King [35]. Complex III (cytochrome c reductase) activity was determined by monitoring the reduction of cytochrome c at 550 nm according to the method of Green and Ziegler. Complex IV (cytochrome c oxidase) activity was evaluated by measuring the oxidation of reduced cytochrome c at 550 nm using the method of Green and Ziegler.

All assays were performed under standardized conditions using freshly prepared reaction mixtures containing the appropriate substrates and cofactors. Enzyme activities were normalized to protein content and expressed as μmol/min/mg protein.

Method S7. Histopathological and Ultrastructural Examination

Kidney tissues intended for histopathological analysis were fixed in 10% neutral-buffered formalin at a ratio of 20:1 for 72 hours. After fixation, samples underwent dehydration via a graded ethanol series, were cleared in xylene, and were subsequently embedded in paraffin wax. Paraffin blocks were sectioned with a rotary microtome to produce 5 μm thick tissue sections, which were subsequently stained with hematoxylin and eosin (H&E) following standard histological protocols. Histopathological alterations were subsequently analyzed using a light microscope.

Renal injury was assessed using a semi-quantitative scoring system in accordance with AASLD guidelines (Table S3), focusing on both tubular and glomerular alterations. Each animal underwent examination of three kidney sections, with four randomly selected microscopic fields analyzed per section at 400× magnification, resulting in a total of 12 fields per animal. Histopathological lesions were classified on a scale of 0 to 3, indicating absent, mild, moderate, and severe changes, respectively. The mean lesion score was computed for each animal and utilized for statistical analysis.

For ultrastructural examination, kidney tissue samples were fixed in 2.5% glutaraldehyde in 0.1 M phosphate buffer at 4 °C for 24 hours and post-fixed in 1% osmium tetroxide for 2 hours. Samples were dehydrated using a graded ethanol series, cleared with acetone, and embedded in epoxy resin. Ultrathin sections measuring 60-70 nm were prepared with an ultramicrotome, subsequently mounted on copper grids, and stained using uranyl acetate and lead citrate. Ultrastructural alterations were analyzed utilizing a JEOL JEM-2100 transmission electron microscope (JEOL, Japan) functioning at 160 kV.

Method S8. Immunohistochemical Assay of NRF2 and NF-κB

Kidney tissue sections embedded in paraffin were deparaffinized using xylene, rehydrated through a graded series of ethanol, and rinsed with distilled water. Antigen retrieval was conducted by heating sections in 0.01 M citrate buffer at pH 6.0 for a duration of 10 minutes. Endogenous peroxidase activity was inhibited by treating the samples with 3% hydrogen peroxide in methanol for 10 minutes, followed by a blocking step with 5% bovine serum albumin (BSA) for 30 minutes at room temperature. Sections were incubated overnight at 4 °C with primary antibodies targeting NRF2 (Abcam, Cambridge, UK; Cat. No. ab62352) and NF-κB p65 (Abcam, Cambridge, UK; Cat. No. ab32536), each at a dilution of 1:200. After PBS washes, sections were incubated with a biotinylated goat anti-rabbit secondary antibody (1:500; Abcam, Cambridge, UK) for 30 minutes at room temperature, followed by a 20-minute incubation with streptavidin–horseradish peroxidase (HRP) conjugate. Immunoreactivity was visualized utilizing 3,3′-diaminobenzidine (DAB) as the chromogen, followed by

counterstaining with hematoxylin. Following dehydration and clearing, sections were mounted using DPX mounting medium.

Immunostained sections were analyzed using a light microscope at a magnification of 400×. Five randomly selected fields from each section were analyzed and photographed utilizing a digital imaging system. Quantitative analysis of positive staining was conducted utilizing ImageJ software, with the results expressed as the percentage of the positively stained area.
